# Assessing the characteristics of MEMS stations for ground-motion recording: A case study of the 2023 M_s_6.2 jishishan earthquake, China

**DOI:** 10.1016/j.isci.2026.116641

**Published:** 2026-07-02

**Authors:** Peibin Xu, Hongwei Wang

**Affiliations:** 1 School of Intelligence and Civil Engineering, Harbin University, Harbin 150086, China; 2 Institute of Engineering Mechanics, China Earthquake Administration, Harbin 150080, China; 3 Key Laboratory of Earthquake Engineering and Engineering Vibration of China Earthquake Administration, Harbin 150080, China

**Keywords:** ground motion characteristics, force-balance accelerometer stations, FBA, micro-electro-mechanical systems stations, MEMS

## Abstract

China established the National Earthquake Intensity Rapid Reporting and Early Warning System (NEIR&EWS) in 2024. The system incorporates a force-balance accelerometer (FBA) and micro-electro-mechanical systems (MEMS) stations. This study systematically evaluated the differences in ground motion intensity measures (IMs) between MEMS and FBA stations using the 2023 Jishishan earthquake as a case study. Recording quality analysis revealed a significantly higher proportion of poor-quality recordings from MEMS stations (21.7%) compared to FBA stations (1.5%). MEMS stations showed a narrower usable period range than FBA stations. Using the normalized log-mean difference method (*Val*_Norm_), we identified significant differences in ground motion intensity measures between station types, with *Val*_Norm_ values exceeding 1 for both horizontal and vertical components under specific conditions. This work serves as a valuable reference for future MEMS data utilization in the NEIR&EWS.

## Highlights

• Micro-electro-mechanical systems (MEMS) stations exhibit lower data quality than FBA stations
• Poor-quality MEMS recordings reach 100% at hypocentral distances beyond 300 km
• MEMS and FBA stations exhibit systematic differences in intensity measures (IMs)
• Wall-mounted MEMS sensors overestimate intensity measures

## Graphical abstract

**Figure:**
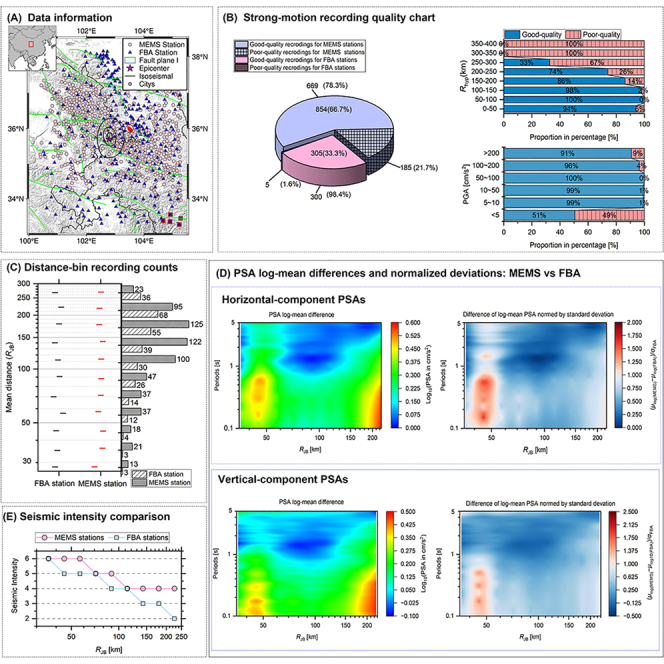

## Teaser

Earth sciences; Geology

## Introduction

Strong motion observation is fundamental to earthquake disaster prevention and earthquake emergency response. Owing to the low cost and fewer deployment constraints, micro-electro-mechanical systems (MEMS) accelerometers are now widely employed for strong-motion observation. These recordings effectively supplement the strong-motion datasets collected by traditional force-balance accelerometer (FBA), supporting the multiple requirements of seismologists and earthquake engineers, particularly in rapid intensity reporting and seismic early warning. Currently, many countries and regions - including Mexico, Italy, New Zealand, Greece and California (United States) - have implemented or are developing seismic early warning systems utilizing MEMS accelerometers.^1^^,^^2^^,^^3^^,^^4^^,^^5^ China had completed the establishment of the National Earthquake Intensity Rapid Reporting and Early Warning System (NEIR&EWS) in 2024. This system comprises 15,899 observation accelerometer stations and significantly enhances nationwide strong-motion recordings collection capabilities. These observation stations in NEIR& EWS are classified into three types^6^: the benchmark station, the basic station, and the general station. Both the benchmark and basic stations used force-balance accelerometer (FBA) as their recording instruments, while the general station deployed a MEMS accelerometer. Therefore, in this study, the benchmark and basic stations were classified as FBA station, whereas the general station was designated as a MEMS station. Table 1 presents the key specifications of MEMS and FBA station, showing MEMS station offer lower dynamic ranges.^7^^,^^8^ Moreover, differences in installation environment requirements and methods existed between the station types. The MEMS stations have less restricted noise requirements for site selection, with the partial equipment installed at a height of no more than 30 cm above the ground. In contrast, FBA stations are subject to strict noise limit standards for site selection, with the equipment installed on a free surface.

**Table 1:** Main technical parameters for MEMS and FBA stations

	Acceleration measurement range	Dynamic range	Measurement error	Operating temperature range
MEMS station	−2.0 g–2.0 g (EW and NS); −2.0 g–2.0 g or -3 g∼3 g (UD)	≥60 dB (0.1 Hz–20 Hz, instrument seismic intensity calculation) ≥80 dB (0.1 Hz–20 Hz, both instrument seismic intensity calculation and earthquake warning)	<5% (0.1 Hz–20 Hz)	−25°C–50°C
FBA station	−2.0 g–2.0 g	≥120 dB	–	−20°C–60°C

Many studies on the performance of MEMS stations have been carried out to examine their feasibility for strong-motion observation. For example, Liu et al.^9^ suggest that the operational environment of MEMS accelerometers is limited and requires minimized vibration interference. The study by Wang and Ding^10^ revealed that MEMS accelerometers have a maximum noise amplitude of about 1 × 10^−2^ ms^−2^, making them unsuitable for monitoring weak vibrations. Consistent result was obtained by Li^11^ their research on earthquake intensity monitoring technology using MEMS accelerometers. Other studies^12^^,^^13^^,^^14^ have revealed that MEMS stations exhibit a narrower effective frequency range. These studies identified significant differences in low-frequency waveforms between MEMS and FBA stations. These phenomena are attributed to differences in instrument performance and installation methods between MEMS and FBA stations. Further extending this line of research, Shi et al.^15^ performed a comparison of the time and frequency-domain differences between MEMS and FBA station to investigate the feasibility of evaluating site conditions with MEMS stations. Wang et al.^16^ systematically quantified the differences between MEMS and FBA stations in both noise characteristics and usable bandwidth within the NEIR&EWS.

Since the operation of the NEIR&EWS, many earthquakes have been well recorded by dense ground-motion observation stations. Many studies investigated ground motion characteristics of earthquake events by integrating these recordings from both MEMS and FBA stations. Such as the 2020 Qiaojia earthquake,^17^ 2020 Guye earthquake,^18^ 2021 Luxian earthquake,^19^ 2021 Yangbi earthquake,^20^ and 2023 Jishishan earthquake^21^^,^^22^ among others. As studies stand at present, most of them on the reliability of MEMS stations have primarily concentrated on the performance and effective frequency band of MEMS accelerometers, while studies on systemic differences in ground motion intensity measures (IMs) remain scarce. Although Qiang et al.^23^ considered differences in ground motion IMs, their comparative analysis focused only on logarithmic deviations of peak ground accelerations (PGAs) and pseudospectral accelerations (PSAs) (at 0.2–4.0 s) between 12 station pairs from the 2021 Yangbi mainshock.

This study aims to systematically evaluate the differences in ground motion IMs between MEMS and FBA stations using the 2023 Jishishan earthquake as a case study. A large number of ground-motion recordings for both MEMS and FBA stations were obtained during this earthquake. These recordings were first examined to reveal their quality. The IMs for horizontal- and vertical-components were then compared to quantitatively evaluate differences between the station types, respectively. The composite three-components IMs were computed to investigate the discrepancies in seismic intensity between MEMS and FBA stations.

## Results

### Comparison of filtering frequencies between MEMS and FBA stations

**Figure 1:**
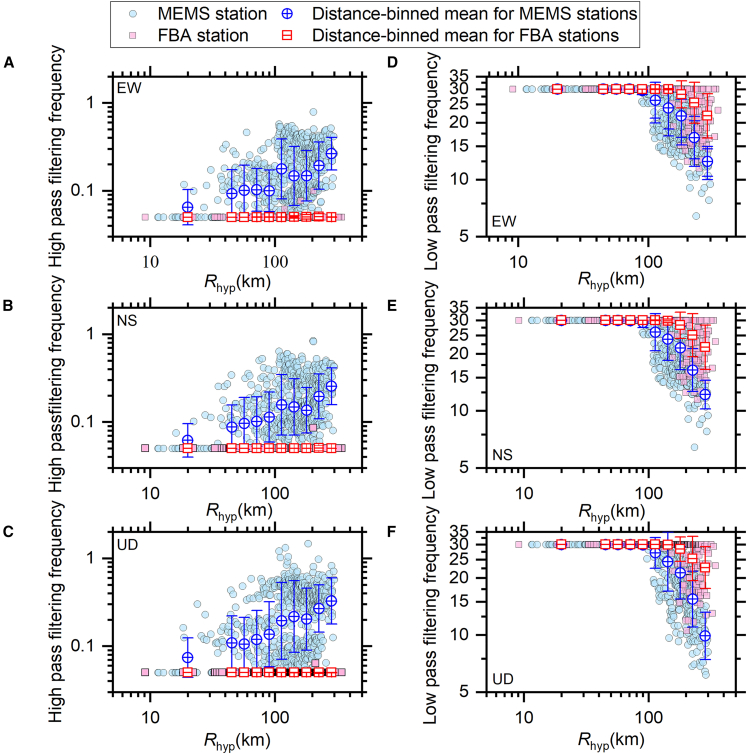
Comparison of filtering frequencies versus hypocentral distance between MEMS and FBA stations High-pass corner frequencies versus hypocentral distance (A) east-west (EW); (B) north-south (NS); and (C) up-down components; low-pass corner frequencies versus hypocentral distance between MEMS and FBA stations: (D) east-west (EW); (E) north-south (NS); and (F) up-down components. The log-mean corner frequencies are calculated in distance bins with a width of 0.1 in log units, and error bars indicate ±1 standard deviation.

A total of 969 good-quality recording was obtained following data processing, of which 300 recordings from FBA stations and 669 from MEMS stations. Figures 1A–1C compare high-pass corner frequencies across all three components (EW, NS, UD) between MEMS and FBA stations versus *R*_hyp_. High-pass corner frequencies ranges for both horizontal and vertical components were as follows: 0.05–0.79 Hz (EW), 0.05–0.85 Hz (NS), and 0.05–1.47 Hz (UD) for MEMS stations; 0.05–0.10 Hz (EW), 0.05–0.09 Hz (NS) and 0.05–0.06 Hz (UD) for FBA stations. The high-pass corner frequencies of MEMS stations for both horizontal and vertical components exhibit greater variability ranges at *R*_hyp_ >30 km, with values trending upward with distance. FBA stations show no significant distance-dependent variability, maintaining a stable corner frequency of 0.05 Hz across the observed distances. Compared to FBAs, MEMS accelerometers have a relatively lower dynamic range (Table 1) and less restricted for site selection requirements (e.g., they can be installed in a communication tower equipment room), which consequently makes their recordings more susceptible to noise than those from FBAs.^13^^,^^16^ Given these characteristics, most MEMS stations exhibited relatively high corner frequencies (0.1–0.8 Hz) in their recordings. The low-pass corner frequencies ranges for the three components (Figures 1D–1F) were as follows: 6.43–30 Hz (EW), 6.71–30 Hz (NS), and 6.15–30 Hz (UD) for MEMS stations, 11.46–30 Hz (EW), 11.96–30 Hz (NS), and 10.52–30 Hz (UD) for FBA stations. For all three components, the low-pass corner frequencies for MEMS stations display significant variability at hypocentral distances exceeding 100 km, with mean values showing a significant decreasing trend. FBA stations show similar result, with apparent variability and a clear decreasing trend in low-pass corner frequencies at longer distances (*R*_hyp_ >200 km). The Figure S1 shows S-wave and noise for MEMS stations located within and beyond 100 km from the hypocenter. The results show that for stations beyond 100 km, there is a clear spectral overlap between the S-wave Fourier spectra and the ambient noise spectra. In contrast, for stations within 100 km, the S-wave spectra are well separated from the noise across the frequency band. These observations explain the low-pass trend (drop beyond 100 km) observed in the data.

### Comparison of number of usable recordings between MEMS and FBA stations

**Figure 2:**
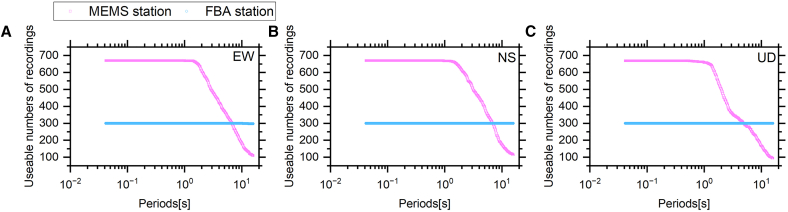
Number of usable recordings versus oscillator period between MEMS and FBA stations (A) east-west (EW), (B) north-south (NS) and (C) up-down components

Figure 2 shows the variation in the number of usable recordings with oscillator period, for three components between MEMS and FBA stations. The upper usable period for PSA was limited to 1.25 times the high-pass corner frequency.^24^ As noted by Douglas and Boore,^25^ there is no inconsistency in the fact that the PSAs at oscillator frequencies near 100 Hz are determined by lower frequencies in the input recordings. Therefore, the number of usable recordings at high frequencies were displayed as uniform. For MEMS stations, the number of usable recordings for both horizontal and vertical components exhibit: (1) a stable plateau at periods <2.0 s, and (2) a steep decreasing trend beyond 2.0 s. The proportion of usable recordings from MEMS stations was greater than 84% at periods of <2.0 s. However, FBA stations show different characteristics, with usable recordings for all three components demonstrating a stable plateau across all periods. The proportion of usable recordings from FBA stations was greater than 84% at periods of <15 s, and exhibited a wider usable period range than that of MEMS stations. The observed differences between station types in Figure 2 may correlate with their deployment standards – FBA stations must satisfy lower level of ambient noise for site selection compared to MEMS stations.

### Horizontal ground motion

In this section, we computed horizontal-component ground motion intensity measures, including significant duration (5%–95%), PGA, peak ground velocity (PGV), and PSAs, to investigate ground motion differences between MEMS and FBA stations during the 2023 Jishishan earthquake.

#### Significant duration analysis

**Figure 3:**
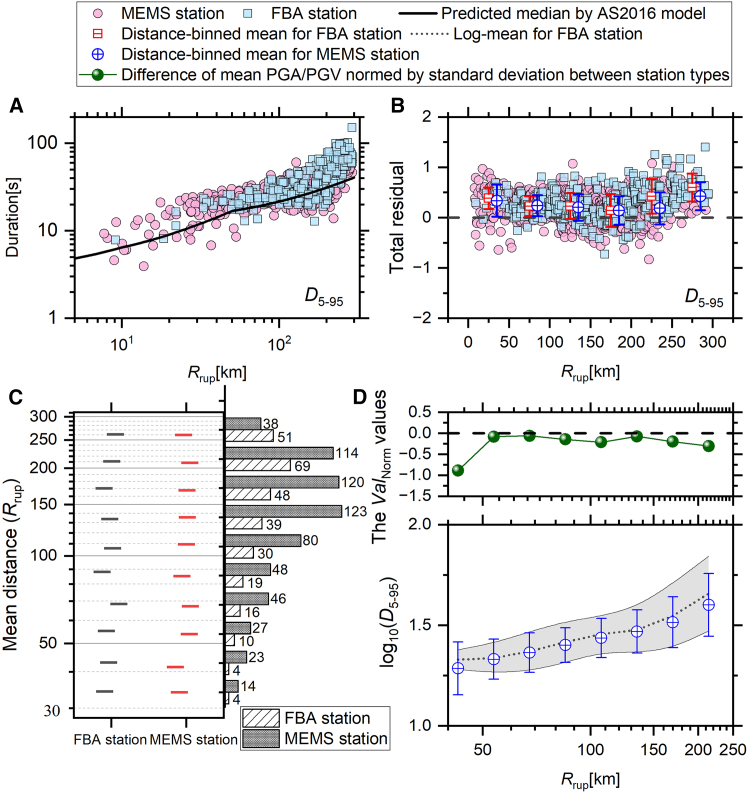
Comparison of the significant duration (*D*_5-95_) between MEMS and FBA (A) Comparison of observed horizontal-component significant duration (5%–95%) between MEMS and FBA stations with the AS2016 model’s median prediction.^27^ (B) The total residuals were calculated using station-specific *V*_S30_ values at each site. Distance-binned mean values of PGA and PGV are presented for: (1) MEMS stations, and (2) FBA station, using 50-unit bin widths. Error bars indicate ±1 standard deviation. (C) The mean *R*_rup_ distance between MEMS and FBA stations is plotted in the left panel, binned in 0.1 log unit intervals from 30 to 300 km. The right panel shows the corresponding distribution of the number of recordings within each distance bin. (D) Comparison of *D*_5-95_ log-mean and standard deviation between MEMS and FBA stations, with the upper panel showing differences of log-mean *D*_5-95_ normalized by the standard deviation of FBA stations.

The significant duration (*D*_5-95_) - defined as the time interval between 5% and 95% of total Arias intensity accumulation^26^ - was calculated for good-quality recordings within *R*_rup_ <300 km from both MEMS and FBA stations. The *D*_5-95_ of the geometrical mean of the two horizontal components for both station types were compared with the ground motion model (GMM) by Afshari and Stewart^27^ (hereafter, AS2016), the *V*_S30_ of which was set to 347 m/s to represent generic soil conditions (Figure 3A). The vast majority of recordings from both MEMS and FBA stations exceeded the AS2016 median predictions, with both station types exhibiting similar distance-dependent amplitude characteristics. Figure 3B presents the total residuals *R*_es_ (computed as the natural log ratio of observed to predicted values) for both MEMS and FBA stations, with mean and ±1 standard deviation at 50 km distance intervals. The *D*_5-95_ predictions at each station were computed using site-specific *R*_rup_ and *V*_S30_ values. Total residuals were predominantly positive for both station types, with minor variations in mean residuals across distance bins.

We calculated log-mean of the IMs values *μ* and the standard deviation of IMs recorded by FBA stations *σ*_FBA_ in 0.1 log-unit distance bins ranging from 30 to 300 km, ensuring at least three recordings per bin and comparable mean distances between the two types (Figure 3C). The smoothing process by the moving window averages for *μ* and *σ*_FBA_ reduces distances ranges and constrains the maximum distance to approximately 240 km. As shown in Figure 3D, the Val_Norm_ values range from −0.062 to −0.889 (upper panel), indicating that the log-mean difference between MEMS and FBA stations ranges from 6.2% to 88.9% of the FBA station’s standard deviation. Observed that the *D*_5-95_ log-mean from MEMS stations were slightly lower than those of FBA stations (lower panel), with log-mean differences (*μ*_log(MEMS)_ minus *μ*_log(FBA)_) ranging from −0.057 to −0.006.

#### PGA、PGV and PSAs analysis

**Figure 4:**
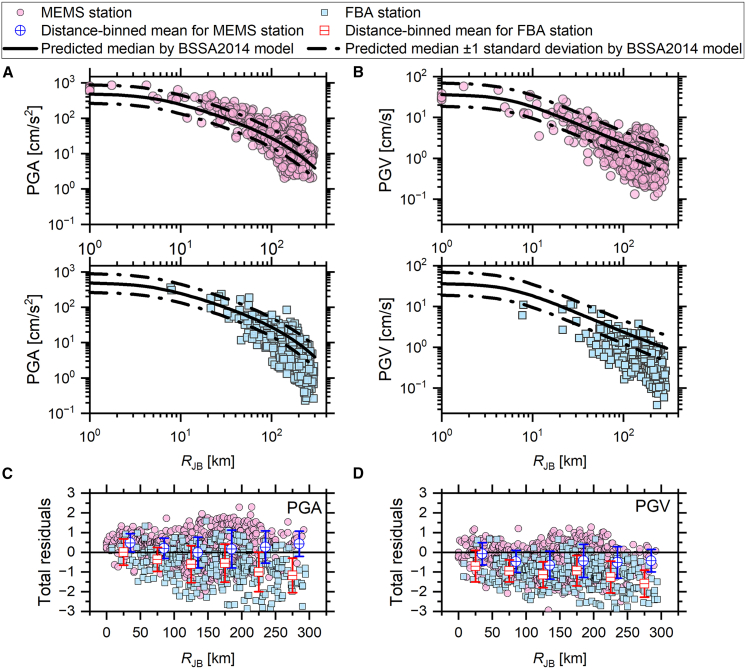
Comparison of PGA and PGV between MEMS and FBA stations Horizontal-component observations of (A) peak ground acceleration (PGA) and (B) peak ground velocity (PGV) plotted against *R*_JB_ for the 2023 Jishishan earthquake, between MEMS and FBA stations. Left vertical axis symbols (*R*_JB_ = 1 km) denote four recordings located at *R*_JB_ = 0 km. Median predictions (solid lines) with ±1 standard deviation ranges (dash-dotted) from BSSA2014 model.^28^ Horizontal-component total residuals of (C) PGA and (D) PGV versus *R*_JB_ for the 2023 Jishishan earthquake, between MEMS and FBA stations. Total residuals were calculated using station-specific *V*_S30_ values at each site. Distance-binned mean values of PGA and PGV are presented for: (1) MEMS stations, and (2) FBA stations, using 50-unit bin widths. Error bars indicate ±1 standard deviation.

Figure 4A present the observed PGA from both MEMS and FBA stations, compared with the GMM by Boore et al.^28^(hereafter, BSSA2014) and ±1*σ*. Recordings from MEMS station predominantly exceed the BSSA14 median prediction, with a substantial proportion surpassing the +1*σ* level. In contrast, recordings from FBA stations predominantly fall below the median prediction, with a subset of observations dropping below the -1*σ* level at *R*_JB_ exceeding 40 km. Many recordings from MEMS stations exhibit higher PGA values than those from FBA stations. The observed PGV were plotted against *R*_JB_ in Figure 4B, along with comparisons to the BSSA2014 model’s median predictions and ±1*σ*. Most recordings from MEMS stations fall below the BSSA2014 median prediction, with numerous observations at *R*_JB_>40 km dropping below the -1*σ* level. For FBA stations, the majority of recordings fall below the median prediction, with an apparent subset dropping below -1*σ* across all distances. Several PGV values recorded by MEMS stations at *R*_JB_>100 km exceeded not only recordings from FBA stations, but also the +1*σ* level of the BSSA2014 model. Figure 4C displays the *R*_es_ for PGA between MEMS and FBA stations, with mean and ±1 standard deviation calculated 50 km distance intervals. Most *R*_es_ for PGA from MEMS stations were positive, while those from FBA stations were predominantly negative. For PGA across all distance intervals, MEMS stations exhibited almost positive mean-*R*_es_ values, while FBA stations showed negative values. A systematic discrepancy in mean-*R*_es_, was observed between station types, with the difference increasing with distance. As shown in Figure 4D, most *R*_es_ for PGV from MEMS stations were negative, whereas those from FBA stations were only predominantly negative. Across all distance bins, both MEMS and FBA stations exhibited negative mean-*R*_es_ values. The two station types showed relatively small differences in mean-*R*_es_ at *R*_JB_<200 km, however, the discrepancy increased markedly when distances exceeded 200 km.

**Figure 5:**
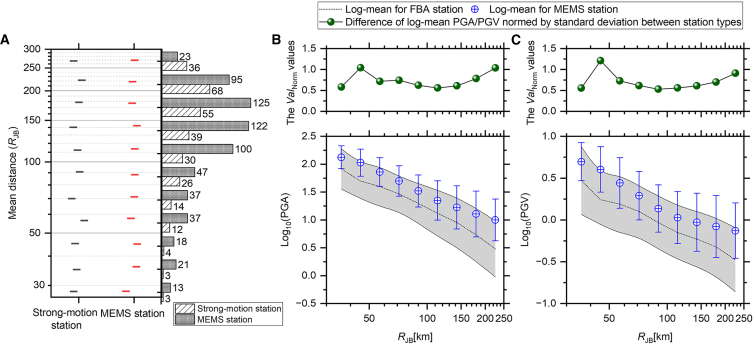
Distance-bin recording counts and PGA/PGV statistics: MEMS vs. FBA (A) The mean *R*_JB_ distance between MEMS and FBA stations is plotted in the left panel, binned in 0.1 log unit intervals from 25 to 300 km. The right panel shows the corresponding distribution of the number of recordings within each distance bin. Comparisons of (B) PGA and (C) PGV log-mean and standard deviation between MEMS and FBA stations, with the upper panel showing differences of log-mean PGA/PGV normalized by the standard deviation of FBA stations.

To quantify the differences for PGA and PGV between MEMS and FBA stations, we computed the *μ* and *σ*_FBA_ in 0.1 log-unit *R*_JB_ distance bins ranging from 25 to 300 km. This range of distances ensures that each distance bin contains at least three recordings and has comparable mean distances between the two station types (Figure 5A). The smoothing process by the moving window averages for *μ* and *σ*_FBA_ reduces distances ranges and constrains the maximum distance to approximately 250 km. As shown in Figures 5B and 5C, the computed *Val*_Norm_ values ranged from 0.558 to 1.042 for PGA and from 0.529 to 1.210 for PGV. The largest *Val*_Norm_ values for PGA were 1.042 at *R*_JB_ of approximately 40∼50 km and 1.037 at *R*_JB_≈200–250 km, and while the largest *Val*_Norm_ value for PGV was 1.210 at *R*_JB_≈40–50 km, indicating differences exceeding 1*σ*_FBA_ between station types. Values exceeding 1.0 (at 40–50 km distances) indicate significant differences between MEMS and FBA stations. For the lower panel, the log-mean across distance bins differed significantly between station types, the log-mean differences between station types ranged from 0.209 to 0.520 for PGA and from 0.168 to 0.362 for PGV, with the values from MEMS stations being higher than those from FBA stations. This result indicated that larger PGA and PGV were recorded by MEMS stations than by FBA stations.

**Figure 6:**
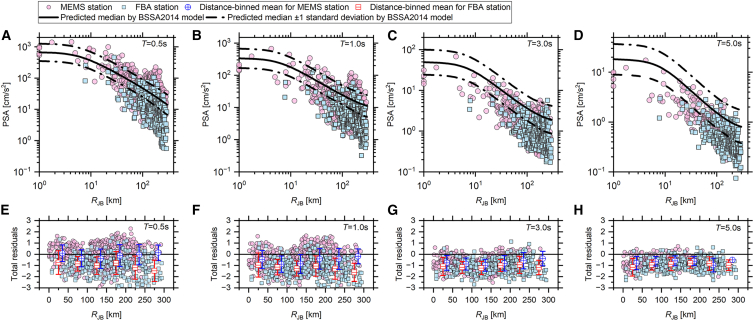
Comparison of PSAs between MEMS and FBA stations Horizontal-component PSAs observations at periods of 0.5 (A), 1.0 (B), 3.0 (C) and 5.0 s (D) plotted against *R*_JB_ for the 2023 Jishishan earthquake, between MEMS and FBA stations. Left vertical axis symbols (*R*_JB_ = 1 km) denote four recordings located at *R*_JB_ = 0 km. Median predictions (solid lines) with ±1*σ* ranges (dash-dotted) from BSSA2014 model.^28^ Horizontal-component total residuals at periods of 0.5 (E), 1.0 (F), 3.0 (G) and 5.0 s (H) versus *R*_JB_ for the 2023 Jishishan earthquake, between MEMS and FBA stations. The total residuals were calculated using station-specific *V*_S30_ values at each recording site. Distance-binned mean values of PSAs at four periods are presented for: (1) MEMS stations, and (2) FBA stations, using 50-unit bin widths. Error bars indicate ±1 standard deviation.

Figures 6A–6D present the PSAs observations at periods of 0.5 s, 1.0 s, 3.0 s and 5.0 s between MEMS and FBA stations, along with the BSSA2014 model’s median predictions and ±1 standard deviation. At period of 0.5 s, most recordings from MEMS station exceeded the BSSA14 median prediction, with a significant portion surpassing the +1*σ* level. Conversely, FBA station recordings primarily fall below the median prediction, particularly at *R*_JB_>40 km where a notable subset falls below the -1*σ* value. At period of 1.0 s, most recordings from both MEMS and FBA stations were lower than the BSSA14 median prediction, with a significant portion falling below the -1*σ* level at *R*_JB_ beyond 40 km. The majority of PSA values for both station types were below the BSSA14 median prediction at periods of 3.0 s and 5.0 s, with many observations under the -1*σ* level. The total residuals of PSAs between MEMS and FBA stations at four periods (0.5, 1.0, 3.0, and 5.0 s) are presented in Figures 6E–6H. The *R*_es_ for PSA from MEMS stations at period of 0.5 s showed fluctuations around zero across distance bins, while the predominantly negative *R*_es_ at FBA stations were observed (Figure 6E). At periods of 1.0 s, 3.0 s, and 5.0 s, observed that most *R*_es_ for PSA for both MEMS and FBA stations were negative (Figures 6F–6H). The discrepancy of the *R*_es_ in PSAs exhibit a systematic reduction with increasing period, especially when *R*_JB_ exceeds 200 km.

**Figure 7:**
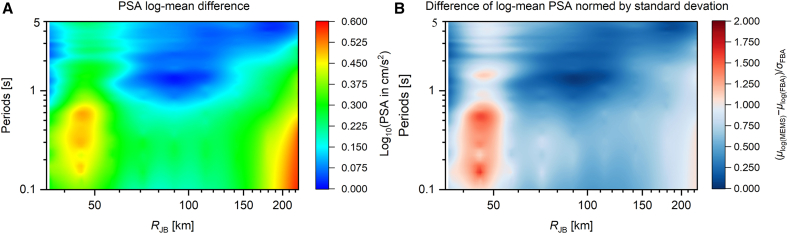
PSA log-mean differences and normalized deviations: MEMS vs. FBA (A) Differences in log-mean horizontal-component PSAs between MEMS and FBA stations across periods of 0.1–5.0 s; (B) Normalized log-mean PSA differences divided by standard deviation of FBA stations across periods of 0.1–5.0 s.

Using distance bins consistent with those for PGA and PGV, the *μ* and *σ*_FBA_ of PSA were calculated at periods of 0.1–5.0 s for MEMS and FBA stations. The log-mean differences between station types ranged from 0.012 to 0.582 across periods of 0.1–5.0 s, with highest values observed at *R*_JB_ exceeding 200 km (Figure 7A). The computed *Val*_Norm_ values ranged from 0.032 to 1.667 across periods of 0.1–5.0 s (Figure 7B). The *Val*_Norm_ values exceeding 1 were predominantly observed in two ranges: (1) at periods of 0.1–1.5 s for near field (*R*_JB_≈40–50 km), and (2) at shorter periods of 0.1–0.5 s for longer distances (*R*_JB_>200 km). These *Val*_Norm_ values reflect discrepancies greater than 1*σ*_FBA_ between station types under these specific conditions.

**Figure 8:**
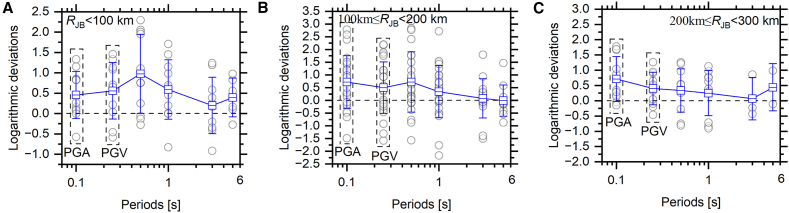
Logarithmic deviations of PGA, PGV, and PSAs by distance range: MEMS vs. neighboring FBA Logarithmic deviations of horizontal-components PGAs, PGVs, and PSAs (at 0.5, 1.0, 3.0, and 5.0 s) between MEMS stations and their neighboring FBA stations for different distance ranges: (A) *R*_JB_ < 100 km, (B) 100 km ≤ *R*_JB_ < 200 km, (C) 200 km ≤ *R*_JB_ < 300 km.

To directly evaluate the performance of the MEMS recordings, we identified 48 station pairs, each consisting of one MEMS and one FBA station, with interstation distances <5 km and azimuth difference <10°. This single-event evaluation eliminates source variability and minimizes path differences, providing an ideal controlled experiment for instrumental comparison. The logarithmic deviations of horizontal-components PGAs, PGVs and PSAs between the MEMS and FBA stations are shown in Figure 8, grouped by Joyner-Boore distance: 11 station pairs for *R*_JB_ < 100 km, 26 station pairs for 100 ≤ *R*_JB_ < 200 km, and 11 station pairs at 200 ≤ *R*_JB_ < 300 km. For each distance group, the majority of station pairs exhibit positive logarithmic deviations, demonstrating that MEMS stations record systematically larger intensity measures than their neighboring FBA stations. To further examine whether elevation differences between neighboring MEMS and FBA stations contribute to the observed discrepancies in ground-motion IMs, we calculated the logarithmic deviation of IMs and performed a linear regression analysis. The absolute elevation difference between each pair was taken as the independent variable. As shown in Figure S2, no statistically significant correlation was found between discrepancies and |ΔElevation| for all considered IMs (all IMs, *p* > 0.05). This result suggests that elevation differences are unlikely to be a cause of the observed IM biases between MEMS and FBA recordings.

### Vertical ground motion

**Figure 9:**
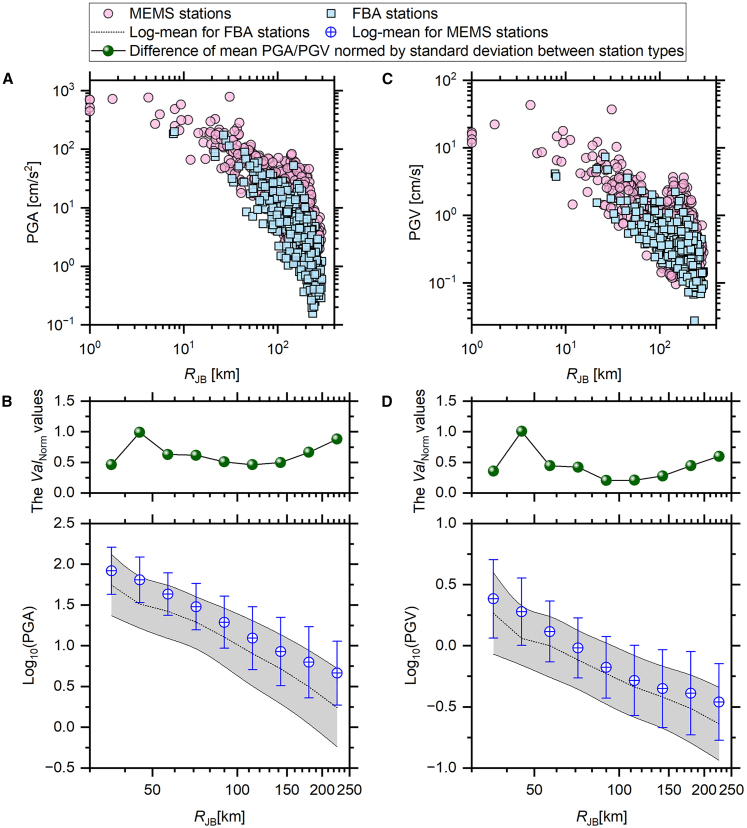
Comparison of PGA and PGV between MEMS and FBA stations Vertical-component observations of ground motion IMs for the 2023 Jishishan earthquake: (A) PGA versus *R*_JB_ for MEMS and FBA stations, with left vertical axis symbols (*R*_JB_ = 1 km) denote four recordings located at *R*_JB_ = 0 km; (B) Log-mean and standard deviation for PGA between MEMS and FBA stations, with the upper panel showing differences of log-mean PGA normalized by the standard deviation of FBA stations; (C) PGV versus *R*_JB_ for MEMS and FBA stations, with left vertical axis symbols (*R*_JB_ = 1 km) denote four recordings located at *R*_JB_ = 0 km; (D) Log-mean and standard deviation for PGV between MEMS and FBA stations, with the upper panel showing differences of log-mean PGV normalized by the standard deviation of FBA stations.

The vertical component of ground motion has been widely recognized as being less susceptible to local site effect.^29^^,^^30^ In order to avoid the effects of site conditions on ground motion, the difference in vertical-component of ground motion IMs (PGA, PGV and PSAs) between MEMS and FBA stations were investigated. To systematically evaluate station-type differences in vertical-component PGA, PGV and PSAs, we computed the *μ* and *σ*_FBA_ using the same distance-binned as applied to the horizontal component. For PGA between station types, similar amplitudes were observed at distance <80 km (*R*_JB_), while a significant proportion of recordings from MEMS station recorded higher PGA compared to FBA stations at *R*_JB_>80 km (Figure 9A). The vertical-component PGAs recorded by FBA stations exhibited faster anelastic attenuation than those from MEMS stations. As shown in Figure 9B, station-type differences in log-mean values remained relatively minimal within 150 km but enlarged substantially at longer distances (*R*_JB_ >150 km). The range of *Val*_Norm_ values for PGA is between 0.462 and 0.991, reflecting a difference of 46.2–99.1%*σ*_FBA_ between station types. The log-mean differences in PGA between station types ranged from 0.173 to 0.423. PGV amplitudes exhibited consistent similarity between station types across all distance range (Figure 9C). The log-mean values exhibited relatively minor differences between station types at *R*_JB_ of 80–150 km but larger differences at other ranges (Figure 9D). The *Val*_Norm_ values for PGV ranged from 0.203 to 1.007, with the value exceeding 1 observed at *R*_JB_ of approximately 40∼50 km, indicating a difference of >1*σ*_FBA_ between station types. For vertical component, it was also observed that the log-mean values for both PGA and PGV were higher at MEMS stations than those at FBA stations. The log-mean differences in PGV between station types ranged from 0.052 to 0.218. This result indicated larger PGA and PGV values at MEMS stations than at FBA stations.

**Figure 10:**
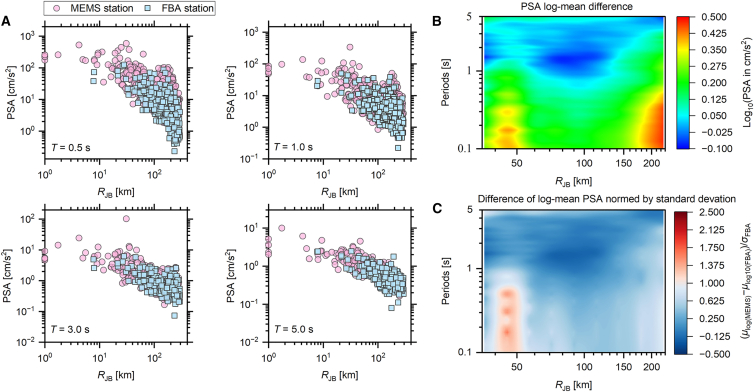
PSA comparison, log-mean differences and normalized deviations: MEMS vs. FBA (A) Vertical-component PSAs observations at periods of 0.5 s, 1.0 s, 3.0 s and 5.0 s plotted against *R*_JB_ for the 2023 Jishishan earthquake, between MEMS and FBA stations. Left vertical axis symbols (*R*_JB_ = 1 km) denote four recordings located at *R*_JB_ = 0 km; (B) Differences in log-mean vertical-component PSAs (0.1–5.0 s) between MEMS and FBA station; (C) Normalized log-mean PSA differences divided by standard deviation of FBA stations across periods of 0.1–5.0 s.

**Figure 11:**
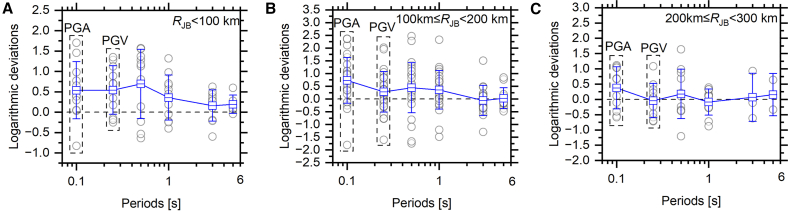
Logarithmic deviations of PGA, PGV, and PSAs by distance range: MEMS vs. neighboring FBA Logarithmic deviations of vertical-component PGAs, PGVs, and PSAs (at 0.5, 1.0, 3.0, and 5.0 s) between MEMS stations and their neighboring FBA stations for different distance ranges: (A) *R*_JB_ < 100 km, (B) 100 km ≤ *R*_JB_ < 200 km, (C) 200 km ≤ *R*_JB_ < 300 km.

The vertical-component PSAs from the Jishishan event were analyzed to quantitatively evaluate the differences between MEMS and FBA stations. Figure 10A plots vertical-component PSAs at specific-periods (0.5 s, 1.0 s, 3.0 s and 5.0 s) versus *R*_JB_. For all four periods, the PSAs displayed comparable amplitudes between station types across all distances. The discrete degree of the PSA amplitudes shows a systematic decrease over comparable distance ranges as the period increases. The log-mean differences in vertical-components PSAs (0.1–5.0 s) between station types ranged from −0.073 to 0.478 (Figure 10B). While differences were relatively minor at *R*_JB_<200 km, they became more pronounced (0.1–0.5 s) at distances exceeding 200 km. The *Val*_Norm_ values for vertical-components PSAs (0.1–5.0 s) ranged from −0.198 to 1.676 (Figure 10C). At near-field region (*R*_JB_≈40–50 km), the *Val*_Norm_ values exceeding 1 for PSA at periods of 0.1–0.6 s, reflecting a difference of >1*σ*_FBA_ between station types. The logarithmic deviations of vertical-component PGAs, PGVs and PSAs between the MEMS and FBA stations (48 station pairs) are also shown in Figure 11, grouped by Joyner-Boore distance intervals of 100 km. For *R*_JB_ <100 km and 100 km ≤ *R*_JB_ < 200 km, the observations show that the majority of pairs are positive, indicating that more intensity measures recorded by MEMS stations are systematically larger than those recorded by the neighboring FBA stations. Similar to the horizontal components, we also calculated the logarithmic deviation of IMs and the absolute elevation differences, and performed a linear regression analysis for the vertical component. As shown in Figure S3, and consistent with the horizontal-component results (all IMs, *p* > 0.05), no statistically significant correlation was found between differences and |ΔElevation| for all considered IMs.

### Seismic intensity

**Figure 12:**
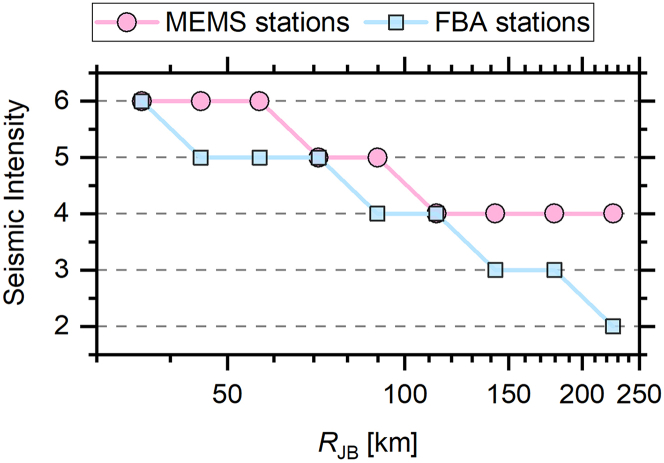
Seismic intensity comparison: MEMS vs. FBA Comparisons of seismic intensity between MEMS and FBA stations were conducted using the moving window average method.

To quantify station-type differences in seismic intensity, the moving window average method uses the same *R*_JB_ bins as those applied to the vertical component IMs when calculating seismic intensity (*I*_s_) statistics. Significant differences in seismic intensity were observed between station types in specific distance-binned (Figure 12). MEMS stations exhibited higher values than FBA stations. The observed differences in *I*_s_ values were 1 at within *R*_JB_ ranges of 40–63 km, 80–100 km, and 125–200 km, and reached up to 2 at *R*_JB_>200 km.

## Discussion

This study presents recordings quality from both MEMS and FBA stations during the 2023 *M*_s_6.2 Jishishan earthquakes. We systematically compared horizontal-component IMs-including *D*_5-95_, PGA, PGV, and PSAs-between station types. Vertical-component IMs were further analyzed to examine station-type differences in attenuation characteristics, along with comparisons of seismic intensity. Our analysis reveals several key findings regarding the reliability of recordings obtained from MEMS stations during the earthquake.

**Figure 13:**
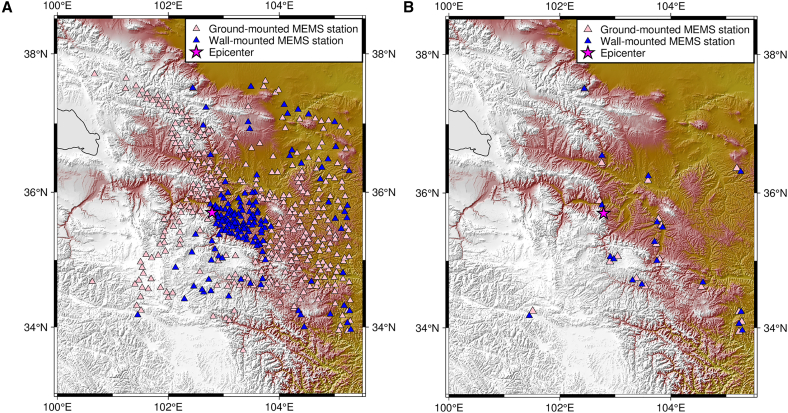
The locations of both ground-mounted and wall-mounted MEMS stations (A) Distribution of known installation mode of MEMS stations that recorded the 2023 Jishishan earthquake is shown as follows: ground-mounted stations are represented by pink triangles, and wall-mounted MEMS stations by blue triangles. (B) Locations of the 18 selected station pairs (≤10 km apart and azimuth difference <10°) used for comparison between ground-mounted and wall-mounted MEMS stations.

1. MEMS stations improved the ability to obtain ground-motion recordings during moderate earthquakes, but exhibited a higher proportion of poor-quality recordings (21.7%) compared to FBA stations (1.5%). The recordings quality from MEMS stations showed a distance-dependent trend (except for *R*_hyp_<50 km). The proportion of poor-quality recordings increases with distance, reaching 100% at hypocentral distances beyond 300 km. Statistical analysis reveals that nearly half (49%) of recordings at PGA<5 cm/s^2^ were identified as poor quality. Additional, FBA stations exhibited wider usable period range than that of MEMS stations. The proportion of usable recordings was greater than 84% for MEMS stations at periods of <2.0 s, and for strong-motion stations at periods of <15 s.
2. The *Val*_Norm_ analysis revealed systematic dependencies between station types for both horizontal and vertical component IMs, with significant differences under specific conditions: For horizontal components IMs, the largest *Val*_Norm_ of *D*_5-95_ reached values of up to −0.889 at *R*_rup_ of 40–50 km, with systematically smaller values observed at MEMS stations than at FBA stations. Conversely, the largest *Val*_Norm_ of PGA was 1.042 at *R*_JB_ distances of 40–50 km, while that of PGV was 1.210 at *R*_JB_≈40–50 km, with MEMS stations consistently exhibiting larger values than FBA stations. The *Val*_Norm_ of PSA exceeded 1 were observed at periods of 0.1–1.5 s in the near field (*R*_JB_ = 40–50 km), and shorter periods of 0.1–0.5 s at longer distances (*R*_JB_≈200–250 km). For vertical-component IMs, the *Val*_Norm_ of both PGA and PGV were larger at MEMS stations than at FBA stations. The largest *Val*_Norm_ of PGA was 0.991 at *R*_JB_≈40–50 km, and the largest *Val*_Norm_ of PGV was 1.007 for near-field recordings (*R*_JB_≈40–50 km). The *Val*_Norm_ of PSA at periods of 0.1–0.6 s exceeded 1 at near field (*R*_JB_≈40–50 km). The significant differences in IMs between station types were smaller for the vertical component than for the horizontal component. This was reflected in the highest *Val*_Norm_, which was reduced by 5% for PGA and 17% for PGV in the vertical component compared to the horizontal component. Otherwise, the significant differences in IMs between two instruments within the 40–50 km distance range may be primarily attributable to the limited number of FBA records available in this bin.
3. Significant differences in seismic intensity were observed between MEMS and FBA stations within specific distance ranges. MEMS stations exhibited higher values, with *I*_s_ differences of 1 within the *R*_JB_ of 40–63 km, 80–100 km and 125–200 km, and difference of up to 2 at *R*_JB_> 200 km. These results indicate that the three-component composite PGA and PGV from MEMS stations was larger than those from FBA stations at specific distance ranges. This observation, along with the larger PGA and PGV values for the horizontal- and vertical-components recorded at MEMS stations, may be attributed to two factors: one factor is the site environments of MEMS stations. They were installed in the equipment room of a communication iron tower, with many situated on hillsides where the topographic amplification effect led to an amplified ground motion. Because the installation method of the MEMS sensors was not disclosed, our analysis was based on the limited installation information that is publicly available for the MEMS stations used in this study. A total of 149 stations were identified where the MEMS sensor is wall-mounted, and 229 stations where the sensor is installed on the ground Figure 13A. It is observed a significant spatial disparity in the distribution between the two groups, as shown by the stations marked with blue triangles (wall-mounted MEMS stations) and pink triangles (ground-mounted MEMS stations).The ideal way to distinguish the influence of different installation modes (ground vs. wall-mounted) is to compare MEMS accelerometers installed at exactly the same site, so that site effects and path effects can be completely excluded. To minimize the influence of site and path effects, we were able to select only 18 neighboring pairs (interstation distance ≤10 km and azimuth difference <10°) between the two installation types (Figure 13B).

**Figure 14:**
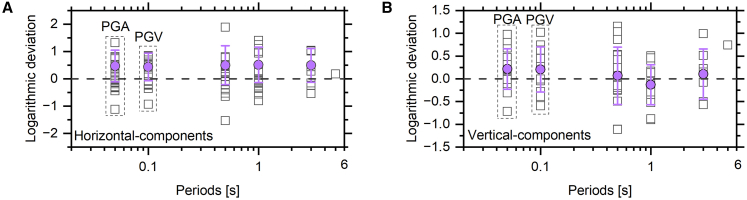
MEMS mounting method comparison Logarithmic deviations of PGA, PGV, and PSA (at 0.5, 1.0, 3.0, and 5.0 s) between wall-mounted and ground-mounted MEMS stations: (A) horizontal component, and (B) vertical component.

For these 18 pairs, we compared the recorded ground motion intensities (PGA, PGV and PSA at 0.5 s, 1.0 s, 3.0 s and 5.0 s) separately for horizontal and vertical components. As shown in Figure 14, we observed that the majority of the differences on a logarithmic scale (wall-mounted minus ground-installed) were positive, suggesting that the wall-mounted installation mode may another factor causing overestimation of ground motion intensity through interaction with the structure.

### Limitations of the study

This study assessed the characteristics of ground-motion recordings from MEMS stations by comparing them with high-performance FBA recordings within the NEIR&EWS in China, based solely on a single event: the 2023 M_s_6.2 Jishishan earthquake. From the perspective of different observation instruments, the findings of this study provide a preliminary reference for the future utilization of MEMS data in NEIR&EWS. Nevertheless, it is important to acknowledge that further investigation incorporating a more diverse collection of ground-motion recordings from more earthquakes is required to determine the generalizability of these findings to events with different magnitudes, source mechanisms, or propagation paths.

## Resource availability

### Lead contact

Further information and requests for resources and materials should be directed to and will be fulfilled by the lead contact, Hongwei Wang (whw1990413@163.com).

### Materials availability

This study did not generate new unique reagents.

### Data and code availability

The strong-motion recordings from MEMS and FBA stations for the 2023 Ms6.2 Jishishan earthquake are provided by the Strong Motion Observation Center, Institute of Engineering Mechanics, China Earthquake Administration. These data are available upon request by contacting csmnc@iem.ac.cn. All data analyses were conducted using publicly available datasets, and both the data processing code and the quantification analysis software are listed in the key resources table. Any additional information required to reanalyze the data reported in this paper is available from the lead contact upon request.

## Acknowledgments

We are grateful to the anonymous reviewers for their constructive comments and suggestions, which greatly improved the quality of this article. This work is supported by the 10.13039/501100012166National Key R&D Program of China (Grant No. 2022YFC3003601), Foundation of Young PhD, Harbin University (Grant No. HUDF2023103).

## Author contributions

Conceptualization, P.B.X. and H.W.W.; methodology, P.B.X. and H.W.W.; investigation, H.W.W.; writing – original draft, P.B.X.; writing – review and editing, P.B.X. and H.W.W.; funding acquisition, P.B.X. and H.W.W.; resources, H.W.W.; supervision, H.W.W.

## Declaration of interests

The authors declare no competing interests.

## STAR★Methods

### Key resources table

**Table:** 

REAGENT or RESOURCE	SOURCE	IDENTIFIER
**Deposited data**		

The raw strong-motion recordings for the 2023 Ms6.2 Jishishan earthquake from MEMS and FBA stations	Strong Motion Observation Center, Institute of Engineering Mechanics, China Earthquake Administration	contact the email csmnc@iem.ac.cn for data application
The processed strong-motion recordings for the 2023 Ms6.2 Jishishan earthquake from MEMS and FBA stations	This manuscript	Zenodo: https://doi.org/10.5281/zenodo.20319766

**Software and algorithms**		

Generic Mapping Tools 6.0	Wessel P, Luis JF, Uieda L et al. (2019). The Generic Mapping Tools Version 6. Geochemistry Geophysics, Geosystem. 20(11): 5556–5564. https://doi.org/10.1029/2019GC008515.	https://www.generic-mapping-tools.org/download/
tspp_documentation_27feb18_version	TSPP	http://www.daveboore.com/
fortran 90	VS code	https://code.visualstudio.com/
Core code	This study	Zenodo: https://doi.org/10.5281/zenodo.20319766

### Experimental model and study participant details

Omitted as our study does not involve biological models.

### Method details

#### Overview of recordings

On 18 December 2023 at 23:59 Beijing Time (15:59 UTC), a magnitude 6.2(*M*_s_) earthquake struck Jishishan County in Linxia Prefecture, Gansu Province, China (China Earthquake Networks Center [CENC], 2023; https://www.cea.gov.cn/cea/dzpd/zqsd/5747510/index.html). This earthquake had a focal depth of 10 km and its epicenter was at 35.700°E, 102.790°N. The epicenter was located along the southeastern segment of the northern margin of Lajishan mountain fault, a major active fault zone that exhibits intense tectonic activity. It was a reverse dip-slip *M*_w_6.0 earthquake according to the focal mechanism solution (strike/dip/rake = 303°/52°/62°) provided by Hua et al.^31^ The maximum intensity reached VIII on the Chinese seismic intensity scale^32^ (equivalent to VIII on the modified Mercalli intensity (MMI) scale), as reported by the China Earthquake Administration (CEA) officially released the seismic intensity map (https://www.mem.gov.cn/xw/yjglbgzdt/202312/t20231222_472849.shtml) (Figure S6).

During the 2023 Jishishan earthquake, a total of 1159 strong-motion recordings were collected within a Joyner-Boore distance (*R*_JB_) of <350 k, 305 from FBA stations and 854 from MEMS stations, representing a significant increase compared to previous earthquakes with similar magnitude. Both MEMS and FBA stations have a sampling rate of 100 samples per second. The MEMS stations were equipped with the MI3000 accelerometer (Beijing Dongli Boyuan Technology Co., Ltd., China) and the REMOS-SIT4 seismometer (Wuhan Seismic Science Instrument Co., Ltd. China). The FBA stations were force-balance accelerometers (±2 g full-scale range), including the ES-T produced by Kinemetrics Inc., and the SLj-100 produced by Institute of Engineering Mechanics, China Earthquake Administration. Practical deployment in most MEMS accelerometer in China being installed in communication tower equipment rooms. There are two installation modes: ground installations and wall-mounted configuration.^16^ As shown in Figure S7, MEMS station N0029 is mounted on the wall, with its sensor housed in the red box. The FBA station is installed in the ground surface observation house, primarily to observe ground motion in free-field soil layers or bedrock surface.

All collected recordings were processed in several steps: The cosine tapers (2.0 s width) were first applied to both the beginning and end of the waveforms to avoid truncation effects, after removing the mean from the entire waveform. The zero pads were appended with sufficient length to beginning and end,^33^ and then 4^th^ -order Butterworth band-pass acausal filter were applied in the frequency domain. Uniform corner frequencies were initially set at 0.05 Hz (high-pass) and 30 Hz (low-pass), with final values determined through based on the signal-to-noise ratio (SNR) and the shape of low-frequency Fourier amplitude spectrum (FAS), as described by Goulet et al.^34^ We compute FAS at frequencies of 0.05–30 Hz for S-wave and pre-P-wave noise windows with the same lengths, and smoothed the spectra to obtained SNR. The S-wave window was extracted based on its onset and end according to Husid plots^35^ and the cumulative root-mean-square function,^36^ respectively. We set the end of noise window at 2.0 s before the P-wave arrival, based on visual inspection of the vertical-component waveform. An SNR threshold of 3.0 was employed in our study. Finally, a total of 190 poor-quality recordings were identified. These poor-quality recordings from MEMS exhibited two issues: truncated waveform tail, as exemplified by station QH.B0030 (hypocentral distance≈29 km; Figure S8A), and noise contamination, as illustrated by station GS.C0008 (hypocentral distance≈298 km; Figure S8B), respectively. Figure S8C presents a pie chart with the proportions of good- and poor-quality recordings for each instrument type (MEMS and FBA stations). The MEMS stations yielded 669 good-quality recordings (78.3%), with the remaining 185 (21.7%) classified as poor-quality, most of which were due to noise-dominated recordings following visual inspection. In contrast, FBA stations produced 300 good-quality recordings (98.4%) and only 5 poor-quality recordings (1.6%) with truncated waveforms. Compared to FBA station, MEMS stations have relatively lower dynamic range and relaxed site requirements, resulting in a relatively high level of noise in their observation recordings, demonstrating that the MEMS stations are more susceptible to environmental noise interference compared to FBA stations.

Figure S8D displays the percentage of good- and poor-quality recordings at MEMS stations, grouped by 50 km *R*_hyp_ intervals. All poor-quality recordings near the epicenter (*R*_hyp_ <50 km) exhibited severely truncated waveform tail, while the remaining recordings at greater distances (*R*_hyp_ >50 km) resulted from noise contamination following visual inspection. The proportion of poor-quality recordings increases with distance across *R*_hyp_ intervals: 2% (100–150 km), 14% (150–200 km), 26% (200–250 km), and 67% (250–300 km), reaching 100% poor-quality recordings at distances beyond 300 km. The recording quality of MEMS stations was statistically analyzed across six PGA interval, as presented in Figure S8E. Poor-quality recordings accounted for 49% at PGA <5 cm/s^2^, 1% in both the 5–10 cm/s^2^ and 10–50 cm/s^2^ ranges, 0% in the 50–100 cm/s^2^ range, 4% in the 100–200 cm/s^2^ range, and 9% at PGA >200 cm/s^2^. All poor-quality recordings in higher PGA ranges (100–200 cm/s^2^ and >200 cm/s^2^) displayed similar characteristics to those of near field recordings, with truncated waveform tails that may be attributed to instrumental causes. Visual inspection confirmed that the remaining poor-quality recordings in all PGA bins below 200 cm/s^2^ resulted from noise contamination. MEMS stations from the NEIR&EWS in China are often deployed in communication tower equipment rooms located in suburban or rural areas, where environmental noise conditions vary significantly.^13^^,^^16^

Two main types of poor-quality recordings are identified in this study, and their causes are as follows. First, under strong shaking conditions in the near field, recordings from MEMS stations at short distances may experience signal interruption and data transmission loss, leading to truncated waveform tails. Second, for stations in the far field, higher noise levels are primarily attributed to the ambient observation environment. In addition, we compared our MEMS recordings with the classification scheme of Wang et al.^16^ Using their recommended PGA thresholds for high-noise instruments, we obtained the consistency index (*I*_*conf*_*)* values of 0.95, 0.89, and 0.84 for the VBBR, BBR, and NBR categories from the bad-quality recordings, respectively (Table S1). These values are similar to those reported in Wang et al.^16^ (approximately 0.99, 0.97, and 0.73). The differences may be explained by the fact that our study is based on a single earthquake while Wang et al.^16^ used a broader multi-event dataset with diverse magnitudes, source mechanisms, and distances.

#### Significant duration

This study used the globally applicable ground motion model (GMM) from AS2016 as a reference to compare significant duration differences between station types. Significant duration in AS2016 model was calculated from geometric mean of the two horizontal components. The primarily predictor variables were constrained as follows: the magnitude ranges were *M*_w_ 3.0–8.0 for strike-slip and reverse-slip earthquakes, and *M*_w_3.0–7.0 for normal-slip earthquakes, with rupture distance (*R*_rup_) of 0–300 km; and the *V*_S30_ (time-weighted average shear-wave velocity over the upper 30 m) values ranging from 150 m/s to 1500 m/s. Therefore, a total of 963 good-quality recordings at *R*_rup_ <300 km were used in this study, including 669 from MEMS stations and 294 from FBA stations. The *V*_S30_ is incorporated into the AS2016 model as the parameter to account for site effects on ground motion characteristics. In this study, the *V*_S30_ values for all stations were derived from a hybrid estimated model integrating surface geological category and subsurface bedrock depth.^37^ The *V*_S30_ values ranged from 189 to 929 m/s for MEMS stations, and from 161 to 883 m/s for FBA stations. According to the National Earthquake Hazard Reduction Program (NEHRP)^38^ classification standards, the site classifications of stations were distributed as follows. The MEMS stations included 5 in class B (760–1500 m/s), 316 in class C (360–760 m/s), and 348 in class D (180–360 m/s). The FBA stations consisted of 1 in class B, 127 in class C, and 162 in class D, and 4 in Class E. Both MEMS and FBA stations exhibited similar site condition distributions, predominantly on NEHRP Class C and D. The median *V*_S30_ values were similar across all datasets: 352 m/s for MEMS stations, 337 m/s for FBA stations, and 347 m/s for the combined dataset. Therefore, the median *V*_S30_ of 347 m/s from the combined dataset was used for the AS2016 median predictions in subsequent analyses. The AS2016 predictions were calculated using the expressions and coefficients given in Equations S1–S8 and Table S2.

#### The normalized log-mean difference method

To better identify and quantify ground motion differences between station types, this study used the normalized log-mean difference method,^39^ which normalizes the log-mean difference by the reference station’s standard deviation (Equation 1). This is accomplished by comparing the distributions of log_10_(IMs) for a subset of binned data across station types.

(Equation 1) $$Val_{\text{Norm}}=\frac{\mu_{\log\left(\text{MEMS}\right)}-\mu_{\log\left(\text{FBA}\right)}}{\sigma_{\text{FBA}}}$$

where *Val*_Norm_ represents the normalized values, which can reflect the ground motion IM differences between station types; *μ*_log(MEMS)_ is the log-mean of the IMs values from MEMS stations, *μ*_log(FBA)_ is the log-mean of the IMs values from FBA stations. Following Dreiling et al. (2014), |ValNorm| ≥ 1.0 indicates a significant difference (the offset exceeds one standard deviation of the reference FBA station). Both *μ*_log(MEMS)_ and *μ*_log(FBA)_ were calculated based on the moving window average, which averages the data over three distances for the specific period range and assigns the average log_10_(IMs) value to the middle distance. The *σ*_FBA_ refers to the standard deviation of IMs recorded by FBA stations.

#### PGA, PGV and PSAs

The globally applicable GMMs for PGA, PGV and PSAs developed by Boore et al.,^28^ were used to investigate the differences in ground motion between MEMS and FBA stations. The BSSA2014 model was derived from the NGA-West2 database, which primarily consists of recordings from California, Taiwan, Japan, China, Italy, Greece, Türkiye, and Alaska. The limitation of the primarily predictor variables in this model was described as follows: the *M*_w_ ranges from 3.0 to 8.5 for strike-slip and reverse-slip earthquakes, and 3.0 to 7.0 for normal-slip earthquakes; *R*_JB_ ranges from 0 to 400 km; and *V*_S30_ has an applicable range of 150–1500 m/s. The ground motion IMs include RotD50-computed PGA, PGV, and PSAs at periods ranging from 0.01 to 10 s. Since the previous comparison of *D*_5-95_ differences between station types was based on recordings at *R*_rup_ <300 km, we maintained the same dataset in this section. We separately compared the observed ground-motion IMs of horizontal components recorded by MEMS stations and FBA stations with the predicted values from the BSSA2014 model. The predicted medians from the BSSA2014 model, along with ±1 standard deviation (*σ*) were computed using the following condition: (1) The magnitude of *M*_w_ = 6.0 with a reverse-slip fault mechanism, (2) *V*_S30_ = 347 m/s representing generic soil condition (derived from a median of the combined sites considered in this study); (3) *Z*_TOR_ = 0 km (Z_TOR_ is the vertical depth to the rupture surface’s shallowest point); (4) regional anelastic attenuation adjustments for China, excluding basin effects. Notably, these predicted medians serve as standardized benchmarks rather than site-specific predictions, as they were computed under uniform site conditions. Their primary purpose is to systematically compare PGA, PGV and PSA values between MEMS and FBA stations, highlighting differences in ground-motion IMs between these two station types. The BSSA2014 predictions were calculated using the expressions and coefficients given in Equations S9–S16 and Tables S3–S5, respectively.

#### Calculation of seismic intensity

The Chinese Seismic Intensity Scale^32^ establishes the seismic instrumental intensity (*I*_I_) evaluation criteria, which is based on three-component composite PGA and PGV values (Equations 2 and 3). Figure S5 shows the flowchart of the *I*_I_ determination process. The seismic intensity (*I*_s_) was determined by calculating *I*_I_ according to the methodology specified.^32^

(Equation 2) $$I_{A}=3.17\;\log_{10}\left(PGA\right)+6.59$$

(Equation 3) $$I_{V}=3.00\;\log_{10}\left(PGV\right)+9.77$$

### Quantification and statistical analysis

Quantitative differences between MEMS and FBA recordings for PGA, PGV, and PSA were assessed using the normalized log-mean difference method (Equation 1, see method details). All calculations were performed using custom Fortran 90 written in Visual Studio Code. Further statistical details are provided in the method details and figure legends.

## Supplemental information

Document S1. Figures S1–S8, Tables S1–S5, and Equations S1–S16
