# Supplementary material for: Assessing the characteristics of MEMS stations for ground-motion recording: A case study of the 2023 Ms6.2 jishishan earthquake, China
Source: iScience. 2026 Jul 2;29(7):116641. doi: 10.1016/j.isci.2026.116641 (PMC13355499; doi:10.1016/j.isci.2026.116641)
Supplement: Document S1. Figures S1–S8, Tables S1–S5, and Equations S1–S16 [file mmc1.pdf]

iScience, Volume *29*

Supplemental information

Assessing the characteristics of MEMS stations for ground-motion recording: A case study of the 2023 M_s6.2 jishishan earthquake, China

Peibin Xu and Hongwei Wang

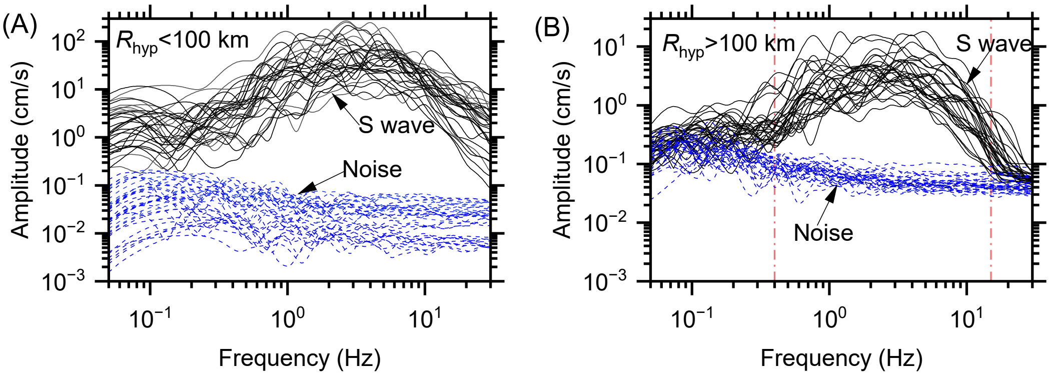

Figure S1. Comparison of Fourier amplitude spectra (FAS) between S-wave and noise for MEMS stations**:** **(a)** R_{hyp}<100 km; and **(b)** R_{hyp}>100 km.

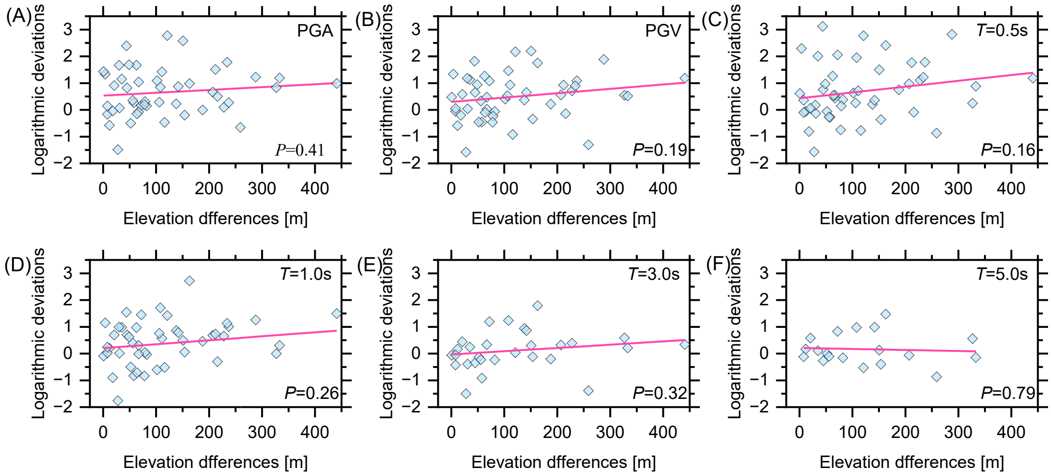

Figure S2. Regression results for the horizontal components between logarithmic deviations and absolute elevation differences for 48 MEMS-FBA station pairs: **(a)** PGA, **(b)** PGV, and PSA at **(c)** 0.5 s, **(d)** 1.0 s, **(e)** 3.0 s, and **(f)** 5.0 s. Each panel shows scatter points and a solid linear regression line.

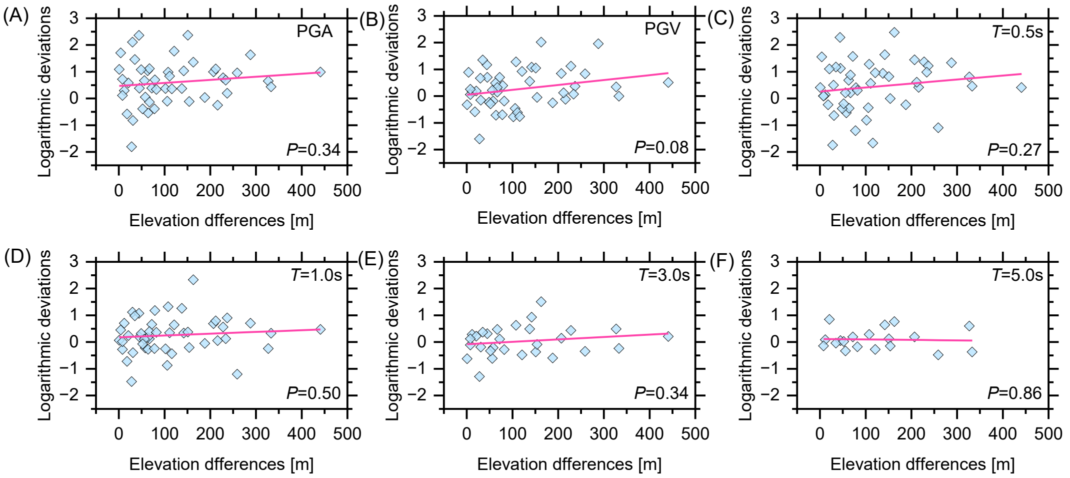

Figure S3. Regression results for the vertical components between logarithmic deviations and absolute elevation differences for 48 MEMS-FBA station pairs: **(a)** PGA, **(b)** PGV, and PSA at **(c)** 0.5 s, **(d)** 1.0 s, **(e)** 3.0 s, and **(f)** 5.0 s. Each panel shows scatter points and a solid linear regression line.

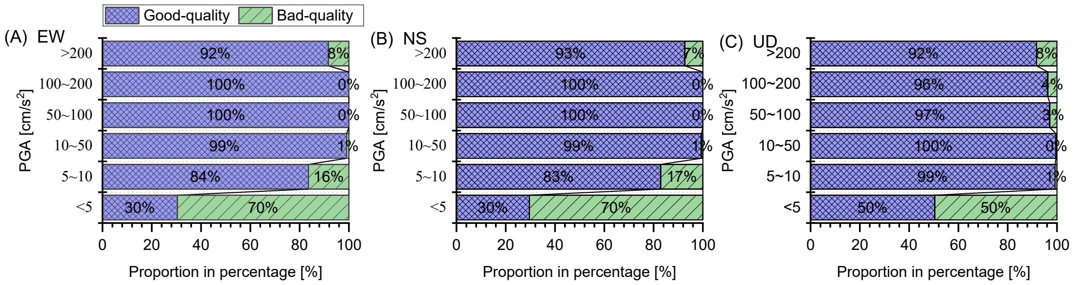

Figure S4. The stacked column graph displays the percentage distribution of good- and poor-quality MEMS station recordings, grouped by six PGA ranges, for each component: **(a)** east-west (EW); **(b)** north-south (NS); and **(c)** up-down (UD).

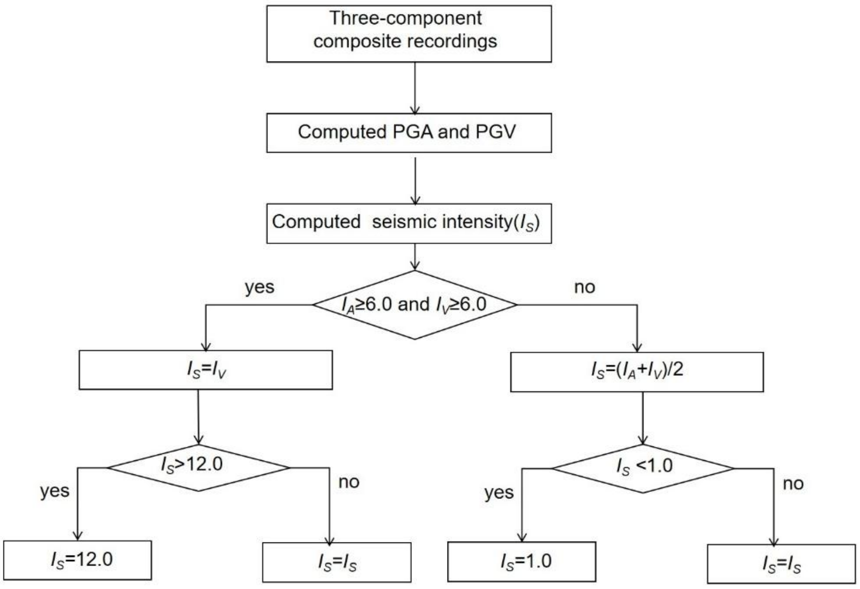

Figure S5. Flowchart of determined seismic instrumental intensity

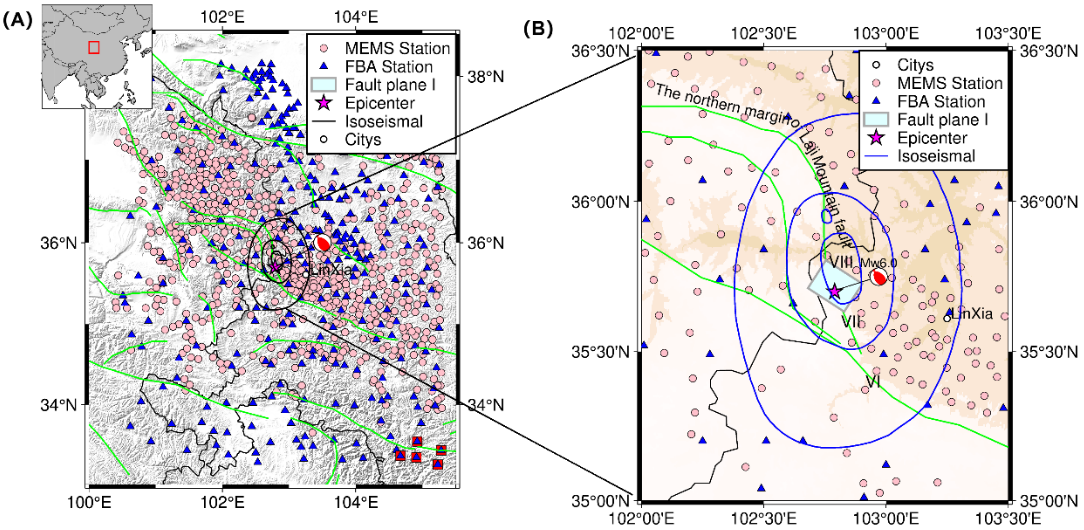

Figure S6. Locations of stations (MEMS and FBA) and earthquake information

(A) Locations of good-quality recordings obtained by stations during the 2023 Jishishan earthquake. MEMS stations are represented by pink circles, while FBA stations are denoted by blue regular triangles. Six triangle symbols marked within a red square were excluded from subsequent analysis due to their rupture distances exceeding 300 km. **(B)** The focal mechanism solution for the 2023 Jishishan earthquake is plotted based on data from Hua et al.[31]. Isoseismal map is from the China Earthquake Administration, with the light-cyan area representing the surface projection of the fault plane by Hua et al.[31].

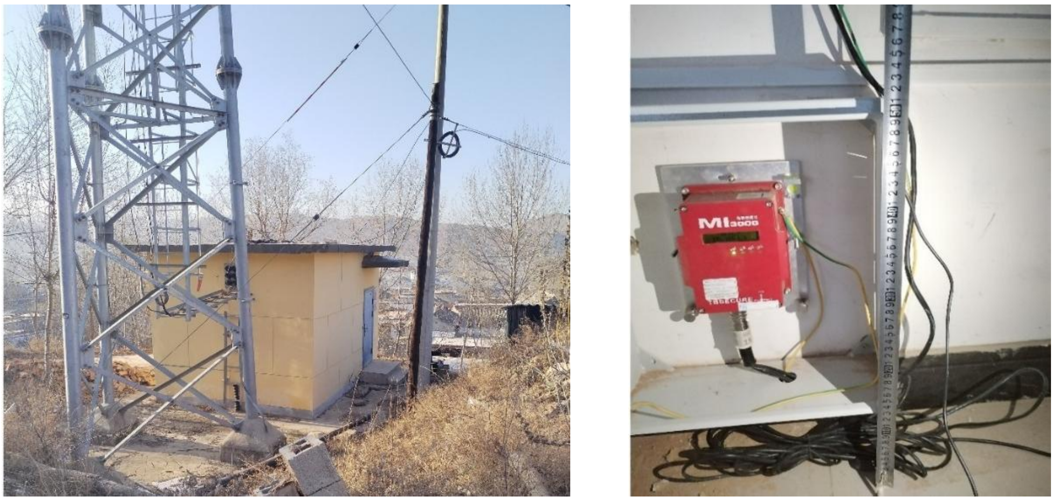

(A) (B)

Figure S7. The station installation environment and internal setup

(A) The external environment surrounding MEMS station N0029 (left panel); **(B)** the internal setup of station N0029, showing the installed MI3000 accelerometer (right panel).

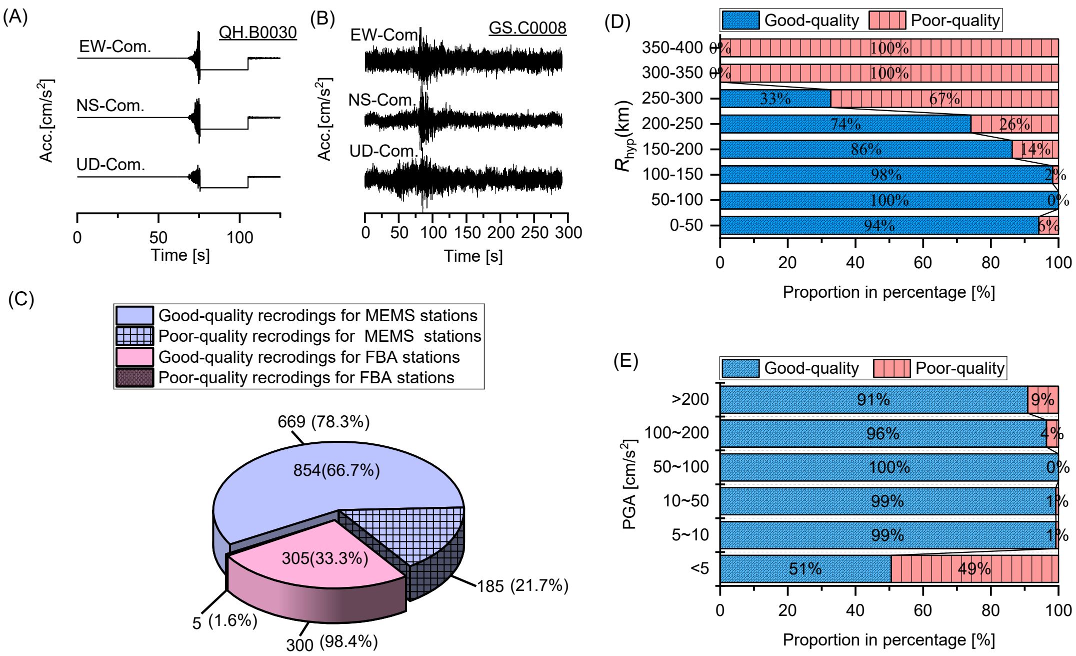

Figure S8. Recording quality chart

The acceleration time series recorded from **(A)** station QH.B0030 and **(B)** station GS.C0008, including the east-west (EW), north-south (NS), and up-down (UD) components. **(C)** Quality distribution of recordings from both MEMS and FBA stations, shown as a pie chart. **(D)** The stacked column graph represents the percentage distribution of good- and poor-quality recording from MEMS stations, grouped by hypocentral distance (R_{hyp}) in 50 km intervals. **(E)** The stacked column graph displays the percentage distribution of good- and poor-quality MEMS station recordings, grouped by 6 PGA ranges. Each component (EW, NS, UD) was displayed in the Supplementary Materials (Figure S4).

Table S1. Summary of MEMS recordings classified by the Wang et al.[16] scheme and the corresponding consistency index (I_{conf})

Classification	Number of recordings	Number of recordings with PGA>PGA_{th}	I_{conf}
VBBR	280	260	0.95
BBR	357	309	0.89
NBR	32	30	0.84

Data S1/Methods S1: Ground motion attenuation relationship models and coefficients used in this study.

$$\ln D = \ln(F_E(M, mech) + F_P(R_{rup})) + F_s(V_{S30}, \delta_{Z_1}) \quad \text{(Equation S1)}$$

In the equation S1, ln$D$ represents the natural logarithm of the significant duration; F_E and F_P denote the source and path duration functions; and F_S is a site term.

$$F_E = \begin{cases} 1/f_0 & M > M_1 \\ b_0(mech) & M \le M_1 \end{cases} \quad \text{(Equation S2)}$$

$$f_0 = 4.9 \bullet 10^6 \beta(\Delta\sigma / M_0)^{1/3} \quad \text{(Equation S3)}$$

Where β is the shear wave velocity at source (3.2km/s), $\Delta\sigma$ is stress drop, and M_0 is the seismic moment in dyne-cm.

$$M_0 = 10^{1.5M+16.05} \quad \text{(Equation S4)}$$

$$\Delta\sigma = \begin{cases} \exp[b_1(mech) + b_2(M - M^*)] & M \le M_2 \\ \exp[b_1(mech) + b_2(M_2 - M^*) + b_3(M - M_2)] & M > M_2 \end{cases} \quad \text{(Equation S5)}$$

where b_1 to b_3, and M_2 are model parameters, and M* denotes a reference magnitude.

$$F_p = \begin{cases} C_1 R_{Rup} & R_{rup} \le R_1 \\ C_1 R_1 + C_2(R_{rup} - R_1) & R_1 \le R_{rup} \le R_2 \\ C_1 R_1 + C_2(R_{rup} - R_1) + C_3(R_{rup} - R_2) & R_{rup} > R_2 \end{cases} \quad \text{(Equation S6)}$$

where C_1 to C_3, R_1 and R_2 are model parameters.

$$F_s = \begin{cases} C_4 \ln(\frac{V_{S30}}{V_{ref}}) + F_{\delta Z_1} & V_{30} \le V_1 \\ C_4 \ln(\frac{V_1}{V_{ref}}) + F_{\delta Z_1} & V_{30} > V_1 \end{cases} \quad \text{(Equation S7)}$$

$$F_{\delta Z_1} = \begin{cases} C_5 \delta Z_1 & \delta Z_1 \le \delta Z_{1,ref} \\ C_5 \delta Z_{1,ref} & \delta Z_1 > \delta Z_{1,ref} \end{cases} \quad \text{(Equation S8)}$$

Where C_4, C_5, V_{ref}, V_1, and $\delta_{z1,ref}$ are model parameters. The AS2016 Model coefficients for reverse earthquakes are given in Tables S2.

Tables S2. Parameters used for reverse earthquakes

	M_1	M_2	M*	b_0(sec)	b_1	b_2	b_3			
Source terms	5.2	7.4	6	1.612	4.536	0.9443	-3.911			
	C_1	C_2	C_3	C_4	C_5	R_1	R_2	V_1	V_{ref}	$V_{Z1,ref}$
Path and site terms	0.3165	0.2539	0.0932	-0.3183	0.0006	10	50	600	369.9	200

$$\ln Y = F_E(M, mech) + F_P(R_{JB}, M, region)) + F_s(V_{S30}, R_{JB}, M, region, Z_1) \quad \text{(Equation S9)}$$

In Equation S9, ln$Y$ represents the natural logarithm of a ground motion IM (PGA, PGV, or PSA); F_E, F_P, and F_S represent source, path, and site effects, respectively.

$$F_E = \begin{cases} a_0 U + a_1 SS + a_2 NS + a_3 RS + a_4(\text{M-M}_h) + a_5(\text{M-M}_h)^2 & M \leq \text{M}_h \\ a_0 U + a_1 SS + a_2 NS + a_3 RS + a_6(\text{M-M}_h) & \text{M>M}_h \end{cases} \quad \text{(Equation S10)}$$

where U, SS, NS, and RS are dummy variables, taking a value of 1 to indicate unspecified, strike-slip, normal-slip, and reverse-slip fault types, respectively, and 0 otherwise; the magnitude M_h is period-dependent, and a_0-a_6 are model coefficients.

$$F_P = [C_1 + C_2(M - M_{ref})]\ln(R / R_{ref}) + (C_3 + \Delta C_3)(R - R_{ref}) \quad \text{(Equation S11)}$$

Where $R = \sqrt{R_{JB}^2 + h^2}$ (Equation S12)

and C_1, C_2, C_3, ΔC_3, M_{ref}, R_{ref} and h are model coefficients. The ΔC_3 is regional adjustments coefficients.

$$F_s = \ln(F_{lin}) + \ln(F_{nl}) \quad \text{(Equation S13)}$$

where F_{lin} and F_{nl} represent the linear and non-linear components of site amplification, respectively.

$$\ln(F_{lin}) = \begin{cases} C\ln(\frac{V_{S30}}{V_{ref}}) & V_{S30} \leq V_C \\ C\ln(\frac{V_C}{V_{ref}}) & V_{S30} > V_C \end{cases} \quad \text{(Equation S14)}$$

where C is model coefficient, V_c is the limiting velocity above which ground motions become independent of V_{S30}, and V_{ref} (set to 760 m/s) is the reference site condition at which the amplification factor equals unity.

$$\ln(F_{nl}) = f_1 + f_2 \ln\frac{PGA_r + f_3}{f_3} \quad \text{(Equation S15)}$$

$$f_2 = f_4[\exp\{f_5(\min(V_{S30}, 760) - 360\} - \exp\{f_5(760 - 360)\}] \quad \text{(Equation S16)}$$

where f_1- f_3 are model coefficients, PGA_r is the median peak horizontal acceleration for reference rock (obtained by evaluating Equation S9 with V_{S30}=760 m/s for a given R_{JB}, M, and *region*), and f_2 represents the degree of nonlinearity as a function of V_{S30}.

Tables S3. Parameters used for source term

period	a_0	a_1	a_2	a_3	a_4	a_5	a_6	M_h
PGV	5.04	5.08	4.85	5.03	1.07	-0.154	0.225	6.2
PGA	0.447	0.486	0.246	0.454	1.43	0.051	-0.166	5.5
0.1	1.13	1.17	0.887	1.15	1.43	0.055	-0.198	5.54
0.11	1.18	1.22	0.927	1.2	1.41	0.037	-0.196	5.57
0.12	1.22	1.26	0.966	1.25	1.38	0.016	-0.193	5.62
0.13	1.26	1.3	1	1.29	1.35	-0.005	-0.189	5.66
0.133	1.27	1.31	1.01	1.3	1.34	-0.011	-0.188	5.67
0.14	1.29	1.33	1.04	1.31	1.32	-0.025	-0.186	5.7
0.15	1.31	1.35	1.06	1.33	1.28	-0.042	-0.182	5.74
0.16	1.32	1.36	1.09	1.34	1.25	-0.058	-0.179	5.78
0.17	1.33	1.37	1.1	1.35	1.22	-0.072	-0.174	5.82
0.18	1.33	1.37	1.11	1.35	1.19	-0.086	-0.169	5.85
0.19	1.33	1.37	1.12	1.35	1.16	-0.099	-0.164	5.89
0.2	1.33	1.36	1.12	1.34	1.13	-0.111	-0.159	5.92
0.22	1.31	1.34	1.11	1.33	1.08	-0.133	-0.147	5.97
0.24	1.29	1.32	1.09	1.31	1.04	-0.153	-0.134	6.03
0.25	1.28	1.3	1.08	1.31	1.02	-0.162	-0.128	6.05
0.26	1.27	1.29	1.07	1.3	0.999	-0.17	-0.121	6.07
0.28	1.24	1.26	1.05	1.28	0.973	-0.185	-0.107	6.11
0.29	1.23	1.25	1.04	1.27	0.963	-0.191	-0.1	6.12
0.3	1.22	1.24	1.02	1.27	0.957	-0.196	-0.093	6.14
0.32	1.2	1.22	1	1.25	0.95	-0.205	-0.079	6.16
0.34	1.18	1.2	0.977	1.23	0.95	-0.211	-0.065	6.18
0.35	1.17	1.18	0.964	1.22	0.951	-0.214	-0.058	6.18
0.36	1.16	1.17	0.951	1.21	0.953	-0.217	-0.051	6.19
0.38	1.13	1.15	0.924	1.18	0.959	-0.222	-0.037	6.19
0.4	1.1	1.12	0.898	1.16	0.968	-0.226	-0.023	6.2
0.42	1.08	1.1	0.871	1.13	0.979	-0.229	-0.01	6.2
0.44	1.05	1.07	0.844	1.1	0.991	-0.232	0.001	6.2
0.45	1.04	1.06	0.829	1.08	0.999	-0.233	0.007	6.2
0.46	1.02	1.04	0.815	1.07	1.01	-0.234	0.012	6.2
0.48	0.997	1.02	0.789	1.04	1.02	-0.235	0.021	6.2
0.5	0.97	0.991	0.762	1.01	1.04	-0.235	0.029	6.2
0.55	0.905	0.928	0.698	0.942	1.08	-0.234	0.047	6.2
0.6	0.842	0.867	0.639	0.874	1.13	-0.231	0.063	6.2
0.65	0.782	0.809	0.582	0.809	1.19	-0.227	0.078	6.2
0.667	0.763	0.79	0.564	0.789	1.2	-0.225	0.083	6.2
0.7	0.725	0.753	0.529	0.75	1.24	-0.221	0.093	6.2

0.75	0.669	0.697	0.475	0.692	1.29	-0.216	0.108	6.2
0.8	0.613	0.642	0.422	0.635	1.33	-0.21	0.123	6.2
0.85	0.559	0.587	0.368	0.58	1.38	-0.205	0.136	6.2
0.9	0.503	0.531	0.314	0.524	1.42	-0.2	0.15	6.2
0.95	0.447	0.475	0.259	0.467	1.46	-0.195	0.164	6.2
1	0.393	0.422	0.207	0.412	1.5	-0.19	0.179	6.2
1.1	0.285	0.314	0.102	0.302	1.57	-0.18	0.21	6.2
1.2	0.173	0.203	-0.006	0.189	1.63	-0.171	0.244	6.2
1.3	0.062	0.091	-0.113	0.074	1.68	-0.162	0.278	6.2
1.4	-0.046	-0.016	-0.216	-0.036	1.72	-0.154	0.31	6.2
1.5	-0.15	-0.119	-0.314	-0.144	1.76	-0.147	0.339	6.2
1.6	-0.249	-0.217	-0.407	-0.247	1.8	-0.14	0.366	6.2
1.7	-0.341	-0.308	-0.493	-0.345	1.83	-0.134	0.391	6.2
1.8	-0.43	-0.396	-0.574	-0.438	1.86	-0.127	0.412	6.2
1.9	-0.513	-0.477	-0.649	-0.527	1.89	-0.12	0.432	6.2
2	-0.587	-0.55	-0.715	-0.607	1.92	-0.112	0.448	6.2
2.2	-0.721	-0.682	-0.83	-0.754	1.97	-0.098	0.48	6.2
2.4	-0.848	-0.807	-0.933	-0.894	2.02	-0.084	0.519	6.2
2.5	-0.91	-0.868	-0.982	-0.962	2.04	-0.076	0.539	6.2
2.6	-0.969	-0.926	-1.03	-1.03	2.06	-0.069	0.558	6.2
2.8	-1.08	-1.04	-1.13	-1.15	2.1	-0.055	0.594	6.2
3	-1.19	-1.14	-1.23	-1.27	2.13	-0.043	0.627	6.2
3.2	-1.29	-1.24	-1.33	-1.38	2.15	-0.034	0.658	6.2
3.4	-1.39	-1.33	-1.42	-1.48	2.17	-0.028	0.688	6.2
3.5	-1.43	-1.38	-1.46	-1.53	2.18	-0.025	0.702	6.2
3.6	-1.48	-1.42	-1.5	-1.58	2.18	-0.023	0.715	6.2
3.8	-1.56	-1.5	-1.59	-1.67	2.19	-0.018	0.74	6.2
4	-1.64	-1.57	-1.67	-1.75	2.2	-0.015	0.763	6.2
4.2	-1.71	-1.64	-1.75	-1.83	2.21	-0.012	0.786	6.2
4.4	-1.78	-1.71	-1.82	-1.9	2.22	-0.012	0.808	6.2
4.6	-1.85	-1.77	-1.89	-1.97	2.22	-0.012	0.831	6.2
4.8	-1.91	-1.83	-1.96	-2.03	2.23	-0.013	0.852	6.2
5	-1.97	-1.89	-2.02	-2.09	2.23	-0.015	0.873	6.2

Tables S4. Parameters used for path term

period	C_1	C_2	C_3	M_{ref}	R_{ref}	h
PGV	-1.24	0.149	-0.00344	4.5	1	5.3
PGA	-1.13	0.192	-0.00809	4.5	1	4.5
0.1	-1.07	0.172	-0.0102	4.5	1	4.13
0.11	-1.06	0.168	-0.00996	4.5	1	4.19
0.12	-1.06	0.164	-0.00972	4.5	1	4.24
0.13	-1.05	0.16	-0.00948	4.5	1	4.29
0.133	-1.05	0.159	-0.0094	4.5	1	4.3

0.14	-1.05	0.157	-0.00923	4.5	1	4.34
0.15	-1.05	0.154	-0.00898	4.5	1	4.39
0.16	-1.05	0.152	-0.00873	4.5	1	4.44
0.17	-1.05	0.149	-0.00847	4.5	1	4.49
0.18	-1.06	0.148	-0.00822	4.5	1	4.53
0.19	-1.06	0.146	-0.00797	4.5	1	4.57
0.2	-1.06	0.145	-0.00772	4.5	1	4.61
0.22	-1.07	0.143	-0.00722	4.5	1	4.68
0.24	-1.07	0.14	-0.00675	4.5	1	4.75
0.25	-1.08	0.139	-0.00652	4.5	1	4.78
0.26	-1.08	0.138	-0.00629	4.5	1	4.82
0.28	-1.09	0.136	-0.00587	4.5	1	4.88
0.29	-1.09	0.135	-0.00567	4.5	1	4.9
0.3	-1.09	0.134	-0.00548	4.5	1	4.93
0.32	-1.1	0.132	-0.00512	4.5	1	4.98
0.34	-1.11	0.13	-0.00481	4.5	1	5.03
0.35	-1.11	0.129	-0.00466	4.5	1	5.06
0.36	-1.11	0.128	-0.00453	4.5	1	5.08
0.38	-1.12	0.126	-0.00428	4.5	1	5.12
0.4	-1.12	0.125	-0.00405	4.5	1	5.16
0.42	-1.13	0.124	-0.00385	4.5	1	5.2
0.44	-1.13	0.123	-0.00367	4.5	1	5.24
0.45	-1.14	0.122	-0.00359	4.5	1	5.25
0.46	-1.14	0.122	-0.00351	4.5	1	5.27
0.48	-1.14	0.121	-0.00336	4.5	1	5.3
0.5	-1.15	0.12	-0.00322	4.5	1	5.34
0.55	-1.15	0.118	-0.0029	4.5	1	5.41
0.6	-1.16	0.117	-0.00261	4.5	1	5.48
0.65	-1.17	0.115	-0.00236	4.5	1	5.53
0.667	-1.17	0.114	-0.00228	4.5	1	5.54
0.7	-1.17	0.113	-0.00213	4.5	1	5.56
0.75	-1.18	0.111	-0.00193	4.5	1	5.6
0.8	-1.18	0.109	-0.00175	4.5	1	5.63
0.85	-1.19	0.107	-0.0016	4.5	1	5.66
0.9	-1.19	0.105	-0.00146	4.5	1	5.69
0.95	-1.19	0.104	-0.00133	4.5	1	5.72
1	-1.19	0.102	-0.00121	4.5	1	5.74
1.1	-1.2	0.1	-0.00099	4.5	1	5.82
1.2	-1.2	0.0985	-0.0008	4.5	1	5.92
1.3	-1.2	0.0974	-0.00064	4.5	1	6.01
1.4	-1.2	0.0967	-0.00049	4.5	1	6.1
1.5	-1.21	0.0964	-0.00037	4.5	1	6.18
1.6	-1.21	0.0963	-0.00026	4.5	1	6.26

1.7	-1.21	0.0963	-0.00017	4.5	1	6.33
1.8	-1.21	0.0962	-0.0001	4.5	1	6.4
1.9	-1.21	0.0963	-0.00004	4.5	1	6.48
2	-1.22	0.0964	0	4.5	1	6.54
2.2	-1.22	0.0965	0	4.5	1	6.66
2.4	-1.22	0.0962	0	4.5	1	6.73
2.5	-1.22	0.0961	0	4.5	1	6.77
2.6	-1.22	0.0961	0	4.5	1	6.81
2.8	-1.22	0.0967	0	4.5	1	6.87
3	-1.22	0.0976	0	4.5	1	6.93
3.2	-1.22	0.0986	-0.00002	4.5	1	6.99
3.4	-1.22	0.0996	-0.00004	4.5	1	7.08
3.5	-1.22	0.1	-0.00005	4.5	1	7.12
3.6	-1.22	0.1	-0.00005	4.5	1	7.16
3.8	-1.22	0.101	-0.00005	4.5	1	7.24
4	-1.22	0.102	-0.00005	4.5	1	7.32
4.2	-1.22	0.103	-0.00005	4.5	1	7.39
4.4	-1.22	0.103	-0.00004	4.5	1	7.46
4.6	-1.22	0.103	-0.00003	4.5	1	7.52
4.8	-1.22	0.103	-0.00001	4.5	1	7.64
5	-1.22	0.104	0	4.5	1	7.78

Tables S5. Parameters used for site term

period	C	V_c	V_{ref}	f_1	f_3	f_4	f_5
PGV	-0.84	1300	760	0	0.1	-0.1	-0.00844
PGA	-0.6	1500	760	0	0.1	-0.15	-0.00701
0.1	-0.487	1479.12	760	0	0.1	-0.249	-0.0056
0.11	-0.506	1474.74	760	0	0.1	-0.252	-0.00562
0.12	-0.524	1469.75	760	0	0.1	-0.255	-0.00567
0.13	-0.542	1464.09	760	0	0.1	-0.256	-0.00572
0.133	-0.548	1457.76	760	0	0.1	-0.257	-0.00574
0.14	-0.56	1450.71	760	0	0.1	-0.257	-0.00578
0.15	-0.58	1442.85	760	0	0.1	-0.257	-0.00585
0.16	-0.601	1434.22	760	0	0.1	-0.256	-0.00591
0.17	-0.623	1424.85	760	0	0.1	-0.254	-0.00597
0.18	-0.645	1414.77	760	0	0.1	-0.252	-0.00602
0.19	-0.667	1403.99	760	0	0.1	-0.249	-0.00608
0.2	-0.688	1392.61	760	0	0.1	-0.247	-0.00614
0.22	-0.724	1380.72	760	0	0.1	-0.242	-0.00626
0.24	-0.756	1368.51	760	0	0.1	-0.238	-0.00638
0.25	-0.772	1356.21	760	0	0.1	-0.236	-0.00644
0.26	-0.787	1343.89	760	0	0.1	-0.233	-0.0065
0.28	-0.816	1331.67	760	0	0.1	-0.226	-0.0066

0.29	-0.83	1319.83	760	0	0.1	-0.223	-0.00665
0.3	-0.842	1308.47	760	0	0.1	-0.219	-0.0067
0.32	-0.862	1297.65	760	0	0.1	-0.213	-0.0068
0.34	-0.877	1287.5	760	0	0.1	-0.208	-0.00689
0.35	-0.884	1278.06	760	0	0.1	-0.206	-0.00693
0.36	-0.89	1269.19	760	0	0.1	-0.204	-0.00697
0.38	-0.9	1260.74	760	0	0.1	-0.2	-0.00705
0.4	-0.911	1252.66	760	0	0.1	-0.196	-0.00713
0.42	-0.922	1244.8	760	0	0.1	-0.192	-0.00719
0.44	-0.935	1237.03	760	0	0.1	-0.187	-0.00726
0.45	-0.941	1229.23	760	0	0.1	-0.185	-0.00729
0.46	-0.947	1221.16	760	0	0.1	-0.183	-0.00732
0.48	-0.959	1212.74	760	0	0.1	-0.179	-0.00738
0.5	-0.969	1203.91	760	0	0.1	-0.175	-0.00744
0.55	-0.989	1194.59	760	0	0.1	-0.166	-0.00758
0.6	-1	1184.93	760	0	0.1	-0.158	-0.00773
0.65	-1.01	1175.19	760	0	0.1	-0.151	-0.00787
0.667	-1.01	1165.69	760	0	0.1	-0.149	-0.00792
0.7	-1.01	1156.46	760	0	0.1	-0.145	-0.008
0.75	-1.02	1147.59	760	0	0.1	-0.139	-0.00812
0.8	-1.02	1139.21	760	0	0.1	-0.132	-0.00822
0.85	-1.03	1131.34	760	0	0.1	-0.125	-0.0083
0.9	-1.04	1123.91	760	0	0.1	-0.118	-0.00836
0.95	-1.04	1116.83	760	0	0.1	-0.112	-0.00841
1	-1.05	1109.95	760	0	0.1	-0.105	-0.00844
1.1	-1.06	1103.07	760	0	0.1	-0.0938	-0.00847
1.2	-1.06	1096.04	760	0	0.1	-0.0841	-0.00842
1.3	-1.06	1088.67	760	0	0.1	-0.0758	-0.00829
1.4	-1.05	1080.77	760	0	0.1	-0.0685	-0.00806
1.5	-1.05	1072.39	760	0	0.1	-0.062	-0.00771
1.6	-1.04	1061.77	760	0	0.1	-0.0559	-0.00723
1.7	-1.04	1049.29	760	0	0.1	-0.0503	-0.00666
1.8	-1.04	1036.42	760	0	0.1	-0.0451	-0.00603
1.9	-1.04	1023.14	760	0	0.1	-0.0404	-0.0054
2	-1.04	1009.49	760	0	0.1	-0.0361	-0.00479
2.2	-1.04	995.52	760	0	0.1	-0.0291	-0.00378
2.4	-1.03	981.33	760	0	0.1	-0.0237	-0.00302
2.5	-1.03	966.94	760	0	0.1	-0.0215	-0.00272
2.6	-1.03	952.34	760	0	0.1	-0.0196	-0.00246
2.8	-1.02	937.52	760	0	0.1	-0.0163	-0.00208
3	-1.01	922.43	760	0	0.1	-0.0136	-0.00183
3.2	-1	908.79	760	0	0.1	-0.011	-0.00167
3.4	-0.995	896.15	760	0	0.1	-0.00867	-0.00158

3.5	-0.991	883.16	760	0	0.1	-0.00757	-0.00155
3.6	-0.987	870.05	760	0	0.1	-0.00654	-0.00154
3.8	-0.978	857.07	760	0	0.1	-0.0047	-0.00152
4	-0.969	844.48	760	0	0.1	-0.00321	-0.00152
4.2	-0.96	832.45	760	0	0.1	-0.0021	-0.00152
4.4	-0.95	821.18	760	0	0.1	-0.00132	-0.0015
4.6	-0.941	810.79	760	0	0.1	-0.000804	-0.00148
4.8	-0.93	801.41	760	0	0.1	-0.000471	-0.00146
5	-0.92	793.13	760	0	0.1	-0.000255	-0.00144
